# Interpretable machine learning-based automated HPLC/MS^2^ platform using ion–molecule reactions for the identification of functionalities in analytes

**DOI:** 10.1039/d5sc07324c

**Published:** 2026-06-02

**Authors:** Armen G. Beck, Ruth O. Anyaeche, Prageeth Wijewardhane, Sanjay Iyer, Yue Fu, Judy Kuan-Yu Liu, Jifa Zhang, Kawthar Z. Alzarieni, Erlu Feng, Ryan T. Hilger, Christopher Welch, Hilkka I. Kenttämaa, Gaurav Chopra

**Affiliations:** a Department of Chemistry, Purdue University 560 Oval Drive West Lafayette IN USA hilkka@purdue.edu gchopra@purdue.edu; b Department of Medicinal Chemistry and Pharmacognosy, Faculty of Pharmacy, Jordan University of Science and Technology P.O. Box 3030, Ar-Ramtha Street Irbid 22110 Jordan; c Department of Computer Science (by courtesy), Purdue Institute for Drug Discovery, Regenstrief Center for Healthcare Engineering, Purdue Center for Cancer Research, Purdue Institute for Inflammation, Immunology and Infectious Disease, Purdue Institute for Integrative Neuroscience West Lafayette IN 47909 USA; d Indiana Consortium for Analytical Science & Engineering (ICASE) Indianapolis Indiana 46202 USA

## Abstract

Identification of unknown compounds in complex mixtures is a time-consuming and challenging problem in several areas of chemistry. High-performance liquid chromatography (HPLC) coupled to tandem mass spectrometry (MS^2^) based on collision-activated dissociation (CAD) is a standard approach used to identify unknown compounds in complex mixtures. However, CAD often produces similar fragmentation patterns for isomeric or related ionized analytes, which makes it difficult to differentiate between similar ions. MS/MS methods based on diagnostic gas-phase ion–molecule reactions provide a powerful, predictable, and reliable alternative for the differentiation of isomeric or similar ions *via* the identification of their specific functional groups. However, the interpretation of the experimental results, the selection of appropriate neutral reagents for new analytes, and the optimization of the conditions for reagent introduction is a manual, time-consuming and challenging process. We have developed a chemical graph-based interpretable machine learning approach that enables automated identification of functionalities in previously unknown protonated analytes, which facilitates the differentiation of isomeric or otherwise similar compounds. Furthermore, this approach significantly advances prior methods used to study ion–molecule reactions by enabling, for the first time, automated selection of neutral reagents for previously unstudied analytes and algorithmic optimization of reagent-dependent pulsing-in and pumping-out times for reagents introduced into the mass spectrometer *via* pulsed valves. This study establishes a foundation for fully automated HPLC/MS^2^ platforms, enabling the differentiation of similar unknown compounds in complex mixtures with broad applications across chemical sciences.

## Abstract 2

An automated HPLC/MS platform for ion–molecule reactions. This platform has been built with interpretable machine learning for analyte identification, reagent selection, and optimized reagent introduction.

## Graphical abstract

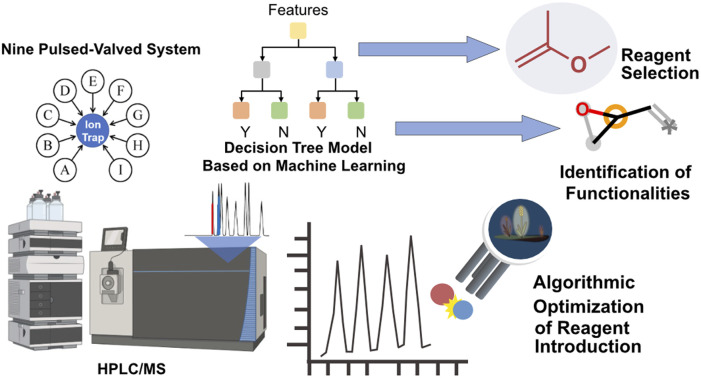

## 1. Introduction

High-performance liquid chromatography (HPLC) coupled with tandem mass spectrometry (MS^2^) based on collision-activated dissociation (CAD) is a widely used technique to obtain structural information for unknown compounds in complex mixtures.^1–4^ However, this technique often produces uninformative data,^5^ usually cannot be used to identify specific functional groups in ionized analytes,^6,7^ and rarely can be used to differentiate isomeric ions.^5,8–10^ Further, examination of several authentic ions is usually required in order to identify an analyte ion. Even then, the identification may fail as many isomeric ions fragment in the same manner and because CAD may cause isomerization. On the other hand, MS^2^ methods based on diagnostic and predictable gas-phase ion–molecule reactions have been utilized successfully to differentiate many isomeric ions and to identify specific functional groups in unknown ionized analytes.^11–19^ Furthermore, this can be done without the aid of standards because ion–molecule reactions are highly predictable after their mechanisms have been delineated. Ion–molecule reactions have been utilized, for example, to differentiate between isomers including isomeric glucuronides^10,20,21^ and to identify *N*-nitrosamines,^19^ carboxylic acids,^12,22^ amides,^13^ and amines.^23^ Most of these experiments involve protonation of the analytes by using ionization techniques such as atmospheric pressure chemical ionization^23^ (APCI) or electrospray ionization (ESI).^6,12^ The protonated analyte molecules are transferred from the ion source into a reaction region, isolated, and allowed to react with neutral reagents. This is usually performed by using ion trap instruments, such as Fourier-transform ion cyclotron resonance (FT-ICR) or quadrupole ion trap mass spectrometers,^17,24^ or triple quadrupole instruments.^25^ Only minor modifications need to be performed on such commercially available instruments.^26^

Gas-phase ion–molecule reactions of protonated compounds with reagents such as tris(dimethylamino)borane (TDMAB), trimethyl borate (TMB), and 2-methoxypropene (MOP) have been studied extensively and can be used for the identification of specific functional groups (sometimes followed by CAD), such as sulfoxide, sulfone, urea, and *N*-oxide.^17,18,27,28^ To achieve this, neutral reagents used in gas-phase ion–molecule reactions can be introduced into a mass spectrometer *via* a continuous flow.^10,18,28^ This approach, however, is limited to the use of one reagent at any given time, preventing high-throughput screening. To address this issue and enable high-throughput screening of complex mixtures, a home-built nine pulsed-valve inlet system has been developed for rapid introduction of several reagents into a linear quadrupole ion trap (LQIT) for ion–molecule reaction studies while the analytes are eluting from an HPLC.^29^ The mass spectrometry experiments can be automated and are fast enough to be performed during HPLC separation.

However, the design of the pulsing sequence for the reagents (the time period during which the reagents are pulsed into the ion trap (pulsing-in time) and the time period during which they are pumped out (pumping-out time)) requires prior knowledge of the appropriate pulsing-in and pumping-out times for each reagent. Furthermore, interpretation of the results and prediction of a neutral reagent for the identification of a new, previously unstudied functionality is challenging. While the method used thus far for the screening of suitable reagents has been effective, it is time-consuming and resource-intensive, requiring consumption of chemicals and solvents. Therefore, implementing an automated system offers a significant advantage by accelerating reagent selection and minimizing experimental waste, while still being informed by the accumulated expert knowledge. To achieve high-throughput screening of analytes and to avoid human bias in the design of the experiments and in the interpretation of the results, we introduce an automated machine-learning (ML) guided HPLC/MS^2^ system to facilitate the identification of functionalities in unknown analytes in complex mixtures. This ML guided HPLC/MS^2^ platform introduces, for the first time, ML automated capabilities for identifying suitable neutral reagents for analytes when no direct structural information is available, optimal selection of reagents, and algorithmic optimization of pulsing-in and pumping-out times, thereby enabling a robust, scalable and automated approach for structural elucidation of compounds in complex mixtures with applications for chemical, biological, and pharmaceutical discovery. Additionally, interpretable ML models, and the learned relations between nontraditional functionalities and reactivity, are introduced for TDMAB and TMB. Overall, this platform allows for the identification of functionalities present in analytes that may not be possible by traditional CAD mediated MS^2^ experiments.

## 2. Experimental section

### 2.1. Materials

Pyridine *N*-oxide (purity 95%), methyl phenyl sulfone (purity ≥98%), phenyl sulfoxide (purity 96%), tris(dimethylamino)borane (purity 99%), and 2-methoxypropene (purity 97%) were obtained from Sigma-Aldrich (Saint Louis, MO, USA). Trimethyl borate (98%) was obtained from Fluka (Buchs, Switzerland). Liquid chromatography/mass spectrometry (LC/MS) grade methanol and water were purchased from Fisher Scientific (Pittsburgh, PA, USA). All chemicals were used as received unless otherwise indicated.

### 2.2. Mass spectrometry instrumentation

All experiments were performed using a slightly modified Thermo Scientific linear quadrupole ion trap (LQIT) mass spectrometer (LTQ XL, Thermo Scientific, San Jose, CA, USA) coupled with an atmospheric-pressure chemical ionization (APCI) source and operated in the positive-ion detection mode. The APCI source conditions were as follows: 300 °C vaporization temperature, 30 (arbitrary units) sheath gas (N_2_) flow rate, 10 (arbitrary units) auxiliary gas (N_2_) flow rate, and 4.0 kV discharge voltage. The capillary voltage was set to 10 V, and the tube lens voltage was set to 40 V. The voltages for the ion optics were optimized using the automated tuning feature of the instrument, LTQ Tune Plus, for the normal mass range from *m*/*z* 50 up to *m*/*z* 500. The instrument was operated using the LTQ Tune Plus interface and Xcalibur 2.2 software. The mass spectrometer was coupled to a Surveyor Plus high-performance liquid chromatograph (HPLC) equipped with a quaternary pump, autosampler, and photodiode array (PDA) detector. The HPLC/MS^2^ experiments were automated using a data-dependent analysis method described in detail below.

Three reagents, 2-methoxypropene, trimethyl borate and tris(dimethylamino)borane (MOP, TMB and TDMAB, respectively), were used in this study. A pulsed valve inlet system was used to introduce each neutral reagent, one after another, as described previously.^29^ The pulsed valve inlet included nine pulsed-valve stems containing two-way tee connectors (Parker Hannifin, Cleveland, OH, USA), a plunger used to isolate each pulsed-valve stem from the instrument, and a variable leak valve (purchased from MKS Instruments, Andover, MA, USA) to introduce a continuous flow of helium through the manifold.^29^ About 5 µL of MOP, TMB, or TDMAB was injected into the different pulsed-valve injection ports by using a 10 µL syringe. A LabVIEW program used to create the pulsing sequence was linked to a high-voltage pulsed-valve driver (built by the Jonathan Amy Facility for Chemical Instrumentation at Purdue), and connected to the nine pulsed valves (Series 9, VAC-1250 PSIG, Parker Hannifin, Cleveland, OH, USA).^29^ The pulsed-valve driver supplied a voltage of up to 300 V to the pulsed valves. Each pulsed valve was either triggered manually or automatically (upon ion isolation) by using the LabVIEW program. The LabVIEW program was used to generate different pulse sequences, *i.e.*, different times for opening the pulsed valves with varying timed delays between the pulses.

### 2.3. High-performance liquid chromatography

The above mass spectrometer was coupled to a Surveyor Plus high-performance liquid chromatograph (HPLC) equipped with a quaternary pump, autosampler, and photodiode array (PDA) detector. For HPLC/MS^2^ analysis, all samples were introduced using an autosampler with a partial loop of 10 µL injection volume. An equimolar mixture containing 100 ppm of each analyte was prepared in methanol. A Zorbax SB-C18 column (4.6 × 250 mm, 5 µm particle size, Agilent Technologies) was used. The mobile phase solvents were pure LC/MS grade water (A) and LC/MS grade methanol (B). The gradient was as follows: 0–5 min isocratic elution at 20% B; 5–15 min linear increase to 95% B, 15–25 min isocratic elution at 95% B, 25–27 linear decreases to 20% B, and 27–30 min isocratic elution at 20% B. Gradient conditions are expressed in terms of the mobile phase B, assuming that the remainder of the composition is mobile phase A. The flow rate was 500 µL min^−1^, and the column temperature was 30 °C. The eluates were subsequently ionized by APCI and transferred into the linear quadrupole ion trap for analysis. The HPLC/MS^2^ experiments were automated using a data-dependent analysis method described in detail below.

### 2.4. Data-dependent analysis

HPLC/MS^2^ experiments were carried out by using the data-dependent scanning feature of Xcalibur 2.2. This feature was used to isolate, one after another, the three most abundant ions detected in the APCI mass spectrum (isolation width 2.0 *m*/*z* units; *q* value 0.25) measured for each eluting compound. This ensured that the protonated analyte was included among the isolated ions, particularly in cases where signals from protonated solvent and solvent related compounds were also present, as solvent-related ions could dominate the spectra. This enabled automated experiments without prior knowledge on the HPLC elution times or MW of the analytes. Additionally, the analytes must produce a signal great enough to overcome noise latently present due to the use of HPLC caused by solvent and other contaminants. A consistent signal-to-noise ratio of at least three was used to identify real signals based on previous work that reports excellent detection limits for this approach (from 50 pM to 250 nM, with the average being 50–100 nM).^30^ The isolated ions were allowed to react for 30 ms with the reagents that were introduced, one after another, into the ion trap *via* the external pulsed-valve inlet system, as described previously.^18^ Given the established behavior of these systems and their known fast reaction kinetics, a reaction time of 30 ms was deemed appropriate and adequate for the current study.^17,18,27,28^ The first ion isolation event triggered the pulsed-valve system. Reagents were pulsed into the ion trap several times until the experiment had completed (see Results and Discussion below). The reagents were introduced sequentially starting with MOP, followed by TMB and finally TDMAB. The pulsing-in time set in the LabVIEW program for all reagents was 150 µs unless otherwise specified. Since the reagents were introduced into the ion trap several times, a 1 s pumping-out time (unless otherwise specified) was used between the closing of a pulsed valve that introduced MOP and opening of another. The pumping-out times for TMB and TDMAB were set at 1.2 s and 2 s, respectively, unless otherwise noted. In the program used to operate the instrument, the charge state of ions for the detection was set to +2. Though ions were singly charged, this setting allowed for the detection of product ions larger than the protonated analyte.^12^

### 2.5. Development and validation of the decision tree models

Decision tree models were generated to identify functional groups in protonated analytes based on whether a diagnostic ion–molecule reaction product was formed or not. For each reagent and diagnostic product type (for MOP, models had been previously published), the available reactions (listed in Tables S6 and S7) were split into a training set and a small external test set (TDMAB: 30 training + 5 test; TMB: 21 training + 5 test). A diverse training set, comprising aliphatic and aromatic mono- and polyfunctional analytes, as well as compounds bearing functionalities structurally related to the target group, was systematically evaluated to probe selectivity and potential interferences. As summarized in Tables S6 and S7, this breadth of chemical space reflects the established approach routinely taken during the development of ion–molecule reaction-based methods to confirm that a reaction can be considered diagnostic. The training set was used for model fitting and internal validation, while the held-out test set was used to assess generalization.

To document the structural diversity of the external test analytes, we computed the average pairwise Tanimoto similarity for each diagnostic product test set (Table S12). The very low similarities observed for the TDMAB models (∼0.065) and low diversity within the TMB models (0.30–0.36) confirm that the held-out analytes span within the studied reaction space, thereby providing a meaningful assessment of model generalization.

All reactions were stoichiometrically balanced. RDKit^31^ was used to convert input SMILES^32^ strings to Morgan fingerprints with a bit length of 2048 and radii of one, two, or three. All input SMILES were canonicalized by RDKit prior to conversion to Morgan fingerprints, ensuring consistent molecular representations across all models. For each reagent and diagnostic product ion, we systematically varied the fingerprint radius and the branching ratio cutoff used to define a “hit” (for example, yield > 0.1), as summarized in Tables S1–S5. Note: The product ion branching ratios correspond to their individual abundances divided by the sum abundance of all product ions. The radius 1 yielded the best performance for the TMB adduct–Me_2_O model, whereas radius 2 was optimal for all remaining diagnostic product ion models, and these radii were used for the final decision trees.

Decision tree models were generated to identify functional groups in protonated analytes based on whether a diagnostic ion–molecule reaction product was formed or not. For each reagent and diagnostic product ion type (for MOP, models have been previously published), the available reactions (listed in Tables S6 and S7) were split into a training set and a small external test set (five reactions) as mentioned before. The training set was used for model fitting and internal validation, while the held-out test set was used to assess generalization.

To quantify the reliability of the model on the training data, we applied leave-one-out cross validation (LOOCV) for each combination of fingerprint radius and branching ratio cutoff. For every left-out reaction, a decision tree was trained on all remaining reactions and used to predict the left-out reaction label. A confusion matrix comparing predicted and true labels was then computed, and the corresponding kappa statistic was used as a measure of agreement beyond chance. This LOOCV procedure was repeated 100 times for each cutoff to account for internal randomness in tree construction, and the maximum kappa value across these repeats was recorded as the primary evaluation metric with *F*1 scores and false discovery rates reported as well (Tables S1–S5).

In addition, for each cutoff, we trained an ensemble of 10 000 decision trees on the training set and used this ensemble to generate predictions on the held-out test set. For every test reaction, the fraction of trees predicting the correct outcome (“formed” or “not formed”) was computed, providing an estimate of predictive consistency on unseen reactions. The entries labelled “compounds 1–5” in Tables S1–S5 report these fractions for each test reaction across branching ratio cutoffs and fingerprint radii. Together, the LOOCV kappa values and the test-set consistency guided the choice of optimal fingerprint radius and branching ratio cutoff for each reagent and diagnostic product.

The distribution of “formed” *versus* “not formed” labels varies across diagnostic product ions, and because this distribution is further influenced by the branching-ratio cutoff used to define a hit (as shown in Tables S1–S5), the kappa statistic provides a more appropriate measure of model performance than raw accuracy, as it accounts for the agreement expected from these changing class proportions.

Once the hyperparameters were selected, a final interpretable decision tree was trained on the full training dataset for each reagent by using the chosen cutoff and fingerprint radius. This step was implemented in the script decision_tree.jl, which also reports 6-fold cross validation accuracy as an additional sanity check. The resulting trees were then analyzed to identify fingerprint bits and corresponding structural motifs that are most predictive of diagnostic reactivity. These motifs were classified as traditional or nontraditional functional groups and used to construct the functional-group identification module and the neutral reagent selection module. Functional groups are classified as traditional or nontraditional based on whether a synthetic organic chemist would typically recognize the corresponding molecular fragment as a known functional group. All machine learning-based decision tree models (Fig. 6b–d) developed for TMB and TDMAB reagents and their identified functional groups are described in the Results and Discussion section. For each reagent, we trained separate decision tree models for each diagnostic product ion channel (for example, for TMB: adduct, adduct–MeOH, and adduct–Me_2_O; for TDMAB: adduct, adduct–DMA, and adduct–2DMA). Each of these diagnostic product ion trees was trained independently on a relevant set of reactions but with a different binary label indicating whether that specific diagnostic product ion was “formed” or “not formed”. In Fig. 6b and d, each dotted box corresponds to one such diagnostic-product–ion specific tree. For visualization, the trees are displayed side-by-side and conceptually combined into a “composite” reagent-level scheme, but in the implementation each diagnostic product tree is used separately when extracting functional group-dependent patterns and making predictions. These extracted groups form the basis for the functional-group identification and reagent selection modules described later in the manuscript.

A single decision tree model was selected rather than ensemble approaches (*e.g.*, Random Forest) because interpretability was a primary objective of this work. A standalone tree provides an explicit, human-readable set of decision rules that can be directly mapped to functional groups and chemical reactivity patterns, enabling mechanistic insight into diagnostic ion–molecule reactions. In contrast, ensemble models average over many trees, making reconstruction of clear functional-group determinants substantially more difficult. Additionally, the decision tree framework maintains continuity with our previously published MOP model and is well matched to the modest dataset size while remaining highly interpretable.^28^

### 2.6. Functional group identification module

High-resolution accurate-mass measurements were combined with an ensemble of bootstrapped decision tree models and expert-based reactivity heuristics for the prediction of the most probable functionalities in unknown analytes. The decision-tree models predict whether a diagnostic ion–molecule reaction product is formed. Functional-group assignments are subsequently obtained by interpreting these predicted reaction outcomes using the extracted structure–reactivity motifs and established ion–molecule reaction rules.

The module used a mass spectrum measured after an ion–molecule reaction, the identity of the reagent used in the experiment, and the *m*/*z*-value of the protonated analyte and its measured elemental composition (performed on a different instrument equipped with a high-resolution Orbitrap mass analyzer) as inputs and predicted plausible functional groups as the output. The mass differences between the *m*/*z* value of the protonated analyte and any detected product ions were determined. A dataset of the determined mass differences and functional groups that could form the detected diagnostic product ion was prepared based on machine learning and experimental data. If a certain mass difference value was not found in the dataset, that value was ignored. Finally, further filtering was carried out using the elemental compositions measured for the protonated analytes (using a different instrument) to filter out functional groups that the analyte could not contain (Fig. 5b). Thus, the machine learning component predicts reaction behavior, while functional-group identification arises from the interpretation of these predictions within the framework of known ion–molecule reactivity.

### 2.7. Reagent selection module

The first step in the design of an experiment that enables successful and rapid identification of functional groups in unknown protonated analytes based on diagnostic ion–molecule reactions is to identify a neutral reagent that reacts with the protonated analyte to form diagnostic products. Therefore, a module was developed for this purpose based on the measured elemental composition of the protonated analyte and its ring and double bond equivalent (RDBE) value. The module contained two methods. One used known and published expert-based data (empirical data) on ion–molecule reactions.^18^ The other method was developed using functionalities identified by machine learning-based decision trees. Both methods in the module used elemental compositions and RDBE values of the protonated analyte as the input to provide a prioritized reagent list as the output (Fig. 6a). If both methods in the script predict the same neutral reagent, then this is ranked to be the first choice for the analyte of interest (Fig. 6b).

### 2.8. Optimization of reagent pulsing-in and pumping-out times

Pulsing-in time is the length of time during which the pulsed valve is open to allow a reagent into the ion trap whereas the pumping-out time is the total time difference between the opening of two pulsed valves. Optimized pulsing-in and pumping-out times, though analyte independent, are reagent dependent and sensitive to day-to-day instrumental/environmental irregularities. To ensure that a reagent is introduced and evacuated effectively, an automated approach for the selection of appropriate pulsing-in and pumping-out times was developed using an in-house metaheuristic package (Paddy).^33^ Paddy was integrated into a graphical user interface (GUI) programmed in Python 3 to facilitate easy accessibility to the full software implementation named Paddy-PUMP. Paddy-PUMP was written with a tkinter front-end user interface and used Paddy for optimization in its back-end code. The pulsing-in and pumping-out times were randomly initiated and allowed to propagate between 70–180 µs and 1–4 s, respectively. Each parameter pair (pulsing-in and pumping-out time combination) was tested in triplicate by introducing the neutral reagent into a mass spectrometer four times and monitoring the length of time needed to pump it out based on the detection of the protonated reagent generated upon reactions with protonated methanol dimers. The resulting data were transferred into the respective extracted ion profiles showing the abundance of the protonated reagent as a function of time. Pulsing-in and pumping-out time parameters were optimized by considering separation quality of extracted ion profile peaks. A peak resolution (*R*) (eqn (1)) of 2.5 was used as the target quantity for sufficient separation between peaks. The first iteration of the optimization was initiated by randomly generating a set of five pairs of pulsing-in and pumping-out times, with subsequent times being dependent on the evaluated performance of the previous iteration.1
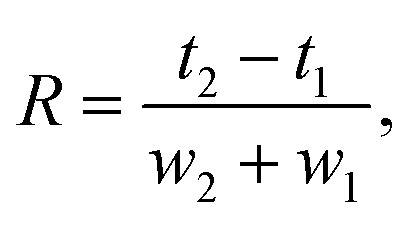
*t* = time point of the peak maximum (min), *w* = width of extracted ion profile peak at half height, *R* = resolution.

Paddy was used to minimize the difference between the average resolution of sets of paired peaks and 2.5, making *R* = 2.5 defined as the maximum of the resulting fitness function (eqn (2)).2
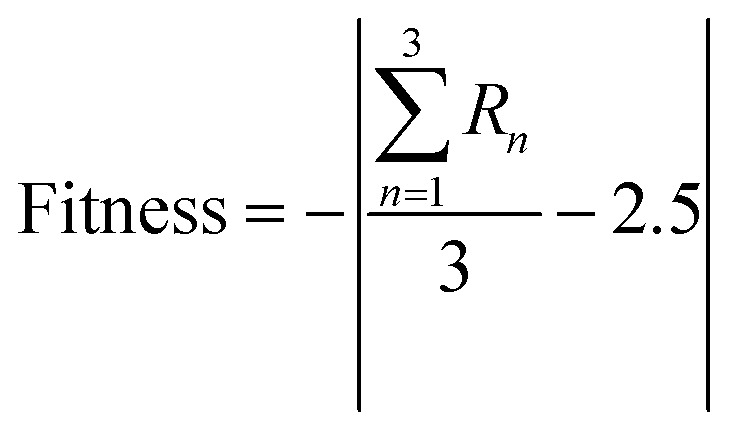


A solution to the optimization problem was defined as a parameter pair generating an average resolution value within ±0.05 of 2.5. Determination of the peak width at half height was performed using the ‘find peaks’ and ‘peak widths’ functions from the ‘signal’ module in the SciPy library (Fig. S1).^34^

With the intent of excluding noise that would otherwise be selected as peaks, a data-driven threshold for peak selection was developed. Gaussian mixture models (GMMs) were employed using the ‘mixture’ module in the Scikit Learn library^35^ to facilitate the data-dependent aspect when calculating the threshold for peak selection. This was accomplished by fitting a GMM with six components to a 2-D list comprised of sorted intensity values and their index, with no regularization of covariance matrices. Once mixture models were fit, they were used to assign extracted ion profile data points to one of the six GMM components (Fig. S2). Of the six subpopulations, the maximal value of the subpopulation with the lowest average abundance value in the extracted ion profile was used to calculate the peak height threshold (eqn (3)).

3 Threshold = 1500 + 2(max(*K*_1_)),

*K*_1_ = subpopulation with lowest average value.

The GUI, Paddy-PUMP, facilitated optimization of reagent introduction by writing ‘recipe’ files for the LabVIEW pulsed-valve driver software in an iterative manner. Recipe files were written such that pulse sequences mirrored the parameters of the pulsing-in and pumping-out times generated by Paddy. Each recipe file was written with five preprogrammed times for a standardized set of reagent injections to initialize the recipe file. This served by providing a signature to denote the start of an experiment in the resulting extracted ion profiles, while also regulating the pulsed-valve system after a period of inactivity (Fig. S1). The five preprogrammed introductions of a reagent were then followed by the corresponding times of the pulsing-in and pumping-out times generated by Paddy for the given iteration. Each parameter pair was written into the recipe file as a quadruplet with a three-second delay between different parameters. Extracted ion profiles were exported from Thermo Xcalibur Qual Browser and subsequently processed by Paddy-PUMP *via* the methods mentioned prior in this section. If no solution was generated by the sequences in the recipe file, fitness values were assigned to the respective parameter pairs and were used to propagate a subsequent iteration. If the peak selection pipeline identified a number of peaks other than a quadruplet per pair of parameters, the fitness value was set to −9 999 999, effectively eliminating it from further propagation. Upon completion of the optimization of pulsing-in and pumping-out times, a textbox notification informed the experimenter that a ‘solutions’ file containing suitable parameters was generated.

## 3. Results and discussion

The goal of this study was to automate a HPLC/MS^2^ system based on diagnostic gas-phase ion–molecule reactions for the rapid identification of functionalities in unknown compounds in mixtures without human interaction. The overview of the machine-learning automated HPLC/MS^2^ system is discussed first, followed by description of the data dependent experiment used to automate the isolation of ionized analytes eluting from HPLC without knowing their retention times or *m*/*z*-values ahead of time. After this, functional-group identification using machine-learning based decision tree model is discussed, followed by the prediction of suitable neutral reagents for new types of analytes. Finally, the optimization of the pulsing-in and pumping-out times of different reagents is discussed.

**Fig. 1:**
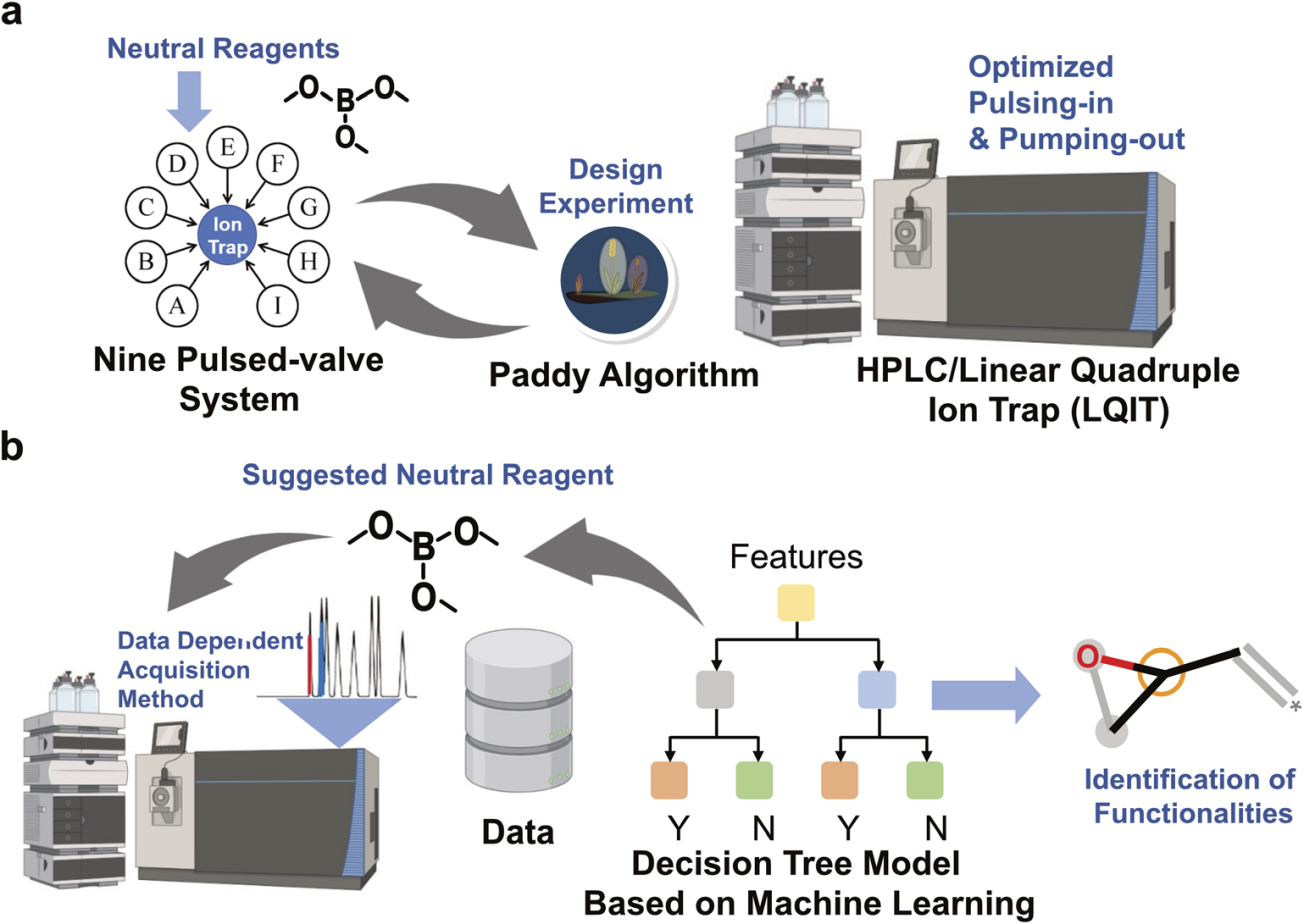
Schematic diagrams showing the overview of the machine-learning automations introduced for HPLC/MS^2^ system based on diagnostic gas-phase ion–molecule reactions for the structural characterization of unknown compounds in mixtures. (a) An evolutionary algorithm, paddy, is used to optimize the pulsing-in and pumping-out times of neutral reagents. (b) Workflows utilizing decision tree models are used for both the identification of functionalities in analytes and the suggestion of neutral reagent for subsequent experimentation.

Ion–molecule reaction experiments began with the introduction of the selected reagents into the nine pulsed-valve inlet system. After this, analytes were injected into the HPLC for separation. A post-column flow splitter was employed to divert the majority of the flow to waste, while approximately 20 µL min^−1^ was directed into the mass spectrometer. The analytes were protonated by APCI as they eluted from the HPLC and then transferred into the ion trap. Data dependent scan methods were used to isolate the three most abundant ions detected in the measured APCI mass spectrum, one after each other, and allowed them to react with reagents introduced, one after each other, from the pulsed-valve system. The generated data were used in the machine learning modules to identify the functionalities in the analytes (Fig. 1). Further, in experiments on previously unknown protonated analytes, the machine learning modules were used to predict suitable neutral reagents (Fig. 1).

### 3.1. Data-dependent experiments

Data-dependent scanning features available on the LQIT mass spectrometer can aid the automation of functional-group identification in ion–molecule reaction experiments and improve rapid screening of unknown analytes. In these experiments, the Xcalibur software algorithms signal the mass spectrometer to perform MS^2^ experiments for the three most abundant ions detected in the initial MS scan (APCI mass spectrum). An overview of the data dependent analysis method is provided first, followed by an example that demonstrates this approach.

**Fig. 2:**
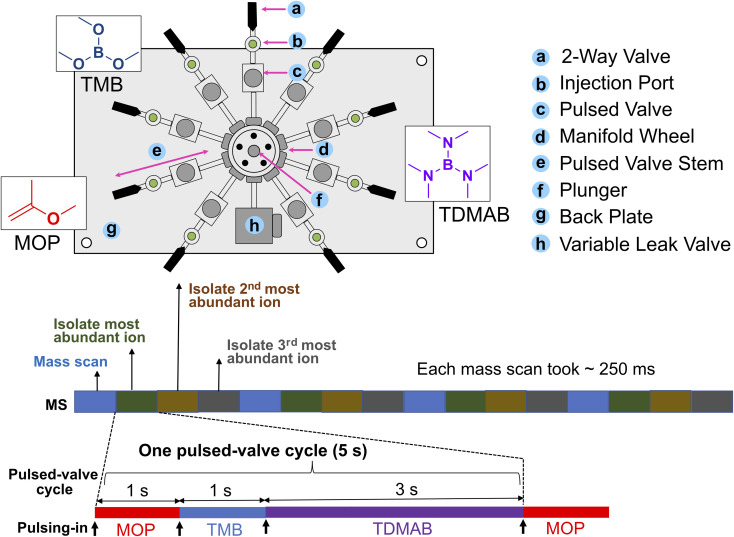
Timeline of one pulsed-valve cycle. An APCI mass spectrum is measured for each eluate. The first ion isolation event triggers the pulsed valve cycle. The three most abundant ions in each APCI mass scan are isolated one after each other, allowed to react with the reagents introduced one after another by the pulsed valve system *via* a variable leak valve, and the products are detected.

**Fig. 3:**
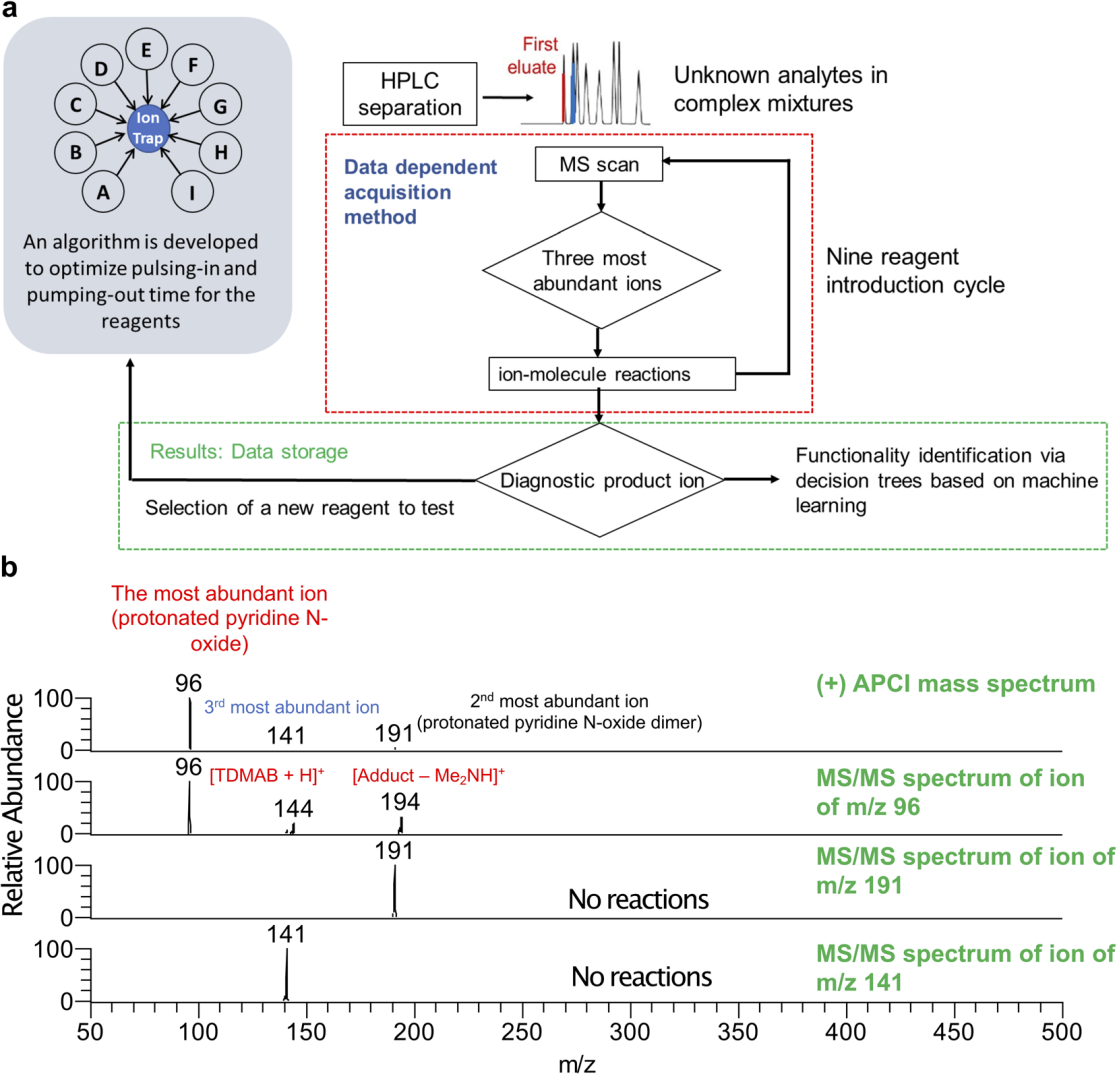
(a) Overview of the automation of the data dependent acquisition method. Unknown analytes in complex mixtures are separated by HPLC and APCI mass spectra are measured for them as they elute from the HPLC. The three most abundant ions in each APCI mass spectrum are isolated, one after another, and allowed to undergo ion–molecule reactions with the neutral reagents introduced by the pulsed valve system one after another. (b) Top: The APCI mass spectrum measured for pyridine *N*-oxide. Below: The MS^2^ mass spectra measured for the three most abundant ions after isolation and reactions with TDMAB for 30 ms.

When performing a HPLC/MS^2^ experiments manually, the unknown mixture first has to be examined by HPLC/MS to determine the elution time and MW of each analyte before manually selecting a MS^2^ program to analyze each analyte. In sharp contrast, an automated experiment based on data dependent scans requires little to no prior knowledge of the analytes, including their retention times or their ions *m*/*z* values. These LC/MS^2^ experiments involved an initial MS scan (Fig. 2) of the eluted analyte to obtain an APCI mass spectrum. The three most abundant ions detected in the APCI mass spectrum are automatically isolated (isolation width of 2 *m*/*z*-units, *q* value 0.25), one after another, by using separate MS^2^ scans (Fig. 2) and allowed react (for 30 ms) with the neutral reagents introduced, one after another, by the pulsed valves (Fig. 3a, S4 and S5).

The LABVIEW program triggers the pulsed-valve system upon the very first ion isolation, which allows the reagents to begin to enter the ion trap from the pulsed valve system, one after another, followed by pumping them out before the next reagent is introduced. The order of the introduced reagents as well as the open time of each pulsed valve (pulsing-in time) and the time delay between two pulses (pumping-out time) determine the pulsing sequence for the neutral reagents. One complete pulsed-valve cycle took approximately five seconds (Fig. 2). This cycle was repeated until the first analyte had eluted from the HPLC. The system automatically paused reagent pulsing until the second analyte began to elute, at which point it automatically re-initiated the sequence, starting with acquisition of an APCI mass spectrum.

An example of the above process is illustrated below for a mixture of pyridine *N*-oxide, methyl phenyl sulfone and diphenyl sulfoxide. The analytes eluted from the HPLC one after the other (Fig. S3), were subjected to APCI, and all ions generated for each analyte were transferred into the ion trap for the measurement of an APCI mass spectrum (Fig. 3b). The three most abundant ions were isolated, one after another, and allowed to react with MOP, TMB and TDMAB introduced from the pulsed valves, one after another (Fig. 3b). The most abundant ions (*m*/*z* 96) generated from the first eluting compound, pyridine *N*-oxide, reacted with TDMAB to form a proton transfer product (*m*/*z* 144) and an adduct that had lost a dimethylamine molecule (*m*/*z* 194). The last product is diagnostic for the *N*-oxide functionality. The other two ions detected in the APCI mass spectrum, those of *m*/*z* 191 and 141, did not react with TDMAB (Fig. 3b). This process was repeated as methyl phenyl sulfone and diphenyl sulfoxide eluted from the HPLC (Fig. S4 and S5). In the case of methyl phenyl sulfone (Fig. S4), the most abundant ions (*m*/*z* 157) reacted with TMB to form a methanol adduct (*m*/*z* 189), an adduct that had lost a dimethyl ether molecule (*m*/*z* 215) and an adduct that had lost a methanol molecule (*m*/*z* 229). The last product is diagnostic for the sulfone functionality. The other two ions detected in the APCI mass spectrum (*m*/*z* 189 and 174) did not react with TMB. For diphenyl sulfoxide (Fig. S5), the most abundant ions (*m*/*z* 203) reacted with MOP to form an adduct (*m*/*z* 275). This product is diagnostic for the sulfoxide functionality. The other two ions detected in the APCI mass spectrum (*m*/*z* 405 and 205) did not react with MOP. Therefore, this experiment enabled the identification of the *N*-oxide, sulfone, and sulfoxide functionalities in the three analytes in one HPLC run.

### 3.2. Functional-group identification module

The functional-group identification module was developed using the decision tree models developed for the three reagents as well as by using experimental data obtained from literature.^18^ The mass spectrum measured after an ion–molecule reaction, the reagent used for the experiment, the measured *m*/*z*-value of the protonated analyte, and the measured elemental composition of the protonated analyte corresponded to the input for the module. The module searches for all the ions above a pre-defined relative abundance cutoff in the ion–molecule reaction mass spectrum and creates a list of *m*/*z* values of the detected ions. Then, the mass differences between the protonated analyte and the detected ion–molecule reaction products are calculated. The module contains a dataset that associates functional groups with specific mass difference values (as explained in section 2.6, Functional group identification module). Finally, the functional groups that are found to correlate with any of the specific mass difference values are selected as the predicted functional groups. Further filtering is performed based on the elemental composition determined for each analyte. However, if the elemental composition of a predicted functional group does not match the elemental composition determined for the unknown analyte, that functional group will be rejected. The functional group prediction module gives two predictions, one based on functionalities identified by the machine learning decision trees, and another based on traditional functional groups (rule-based method) identified using experimental data. Therefore, the rule-based method effectively serves as a built-in baseline. This allows users to directly compare mass-difference rules with the decision tree predictions where the decision tree-identified structural motifs provide additional discriminatory power beyond simple mass-difference matching.

**Fig. 4:**
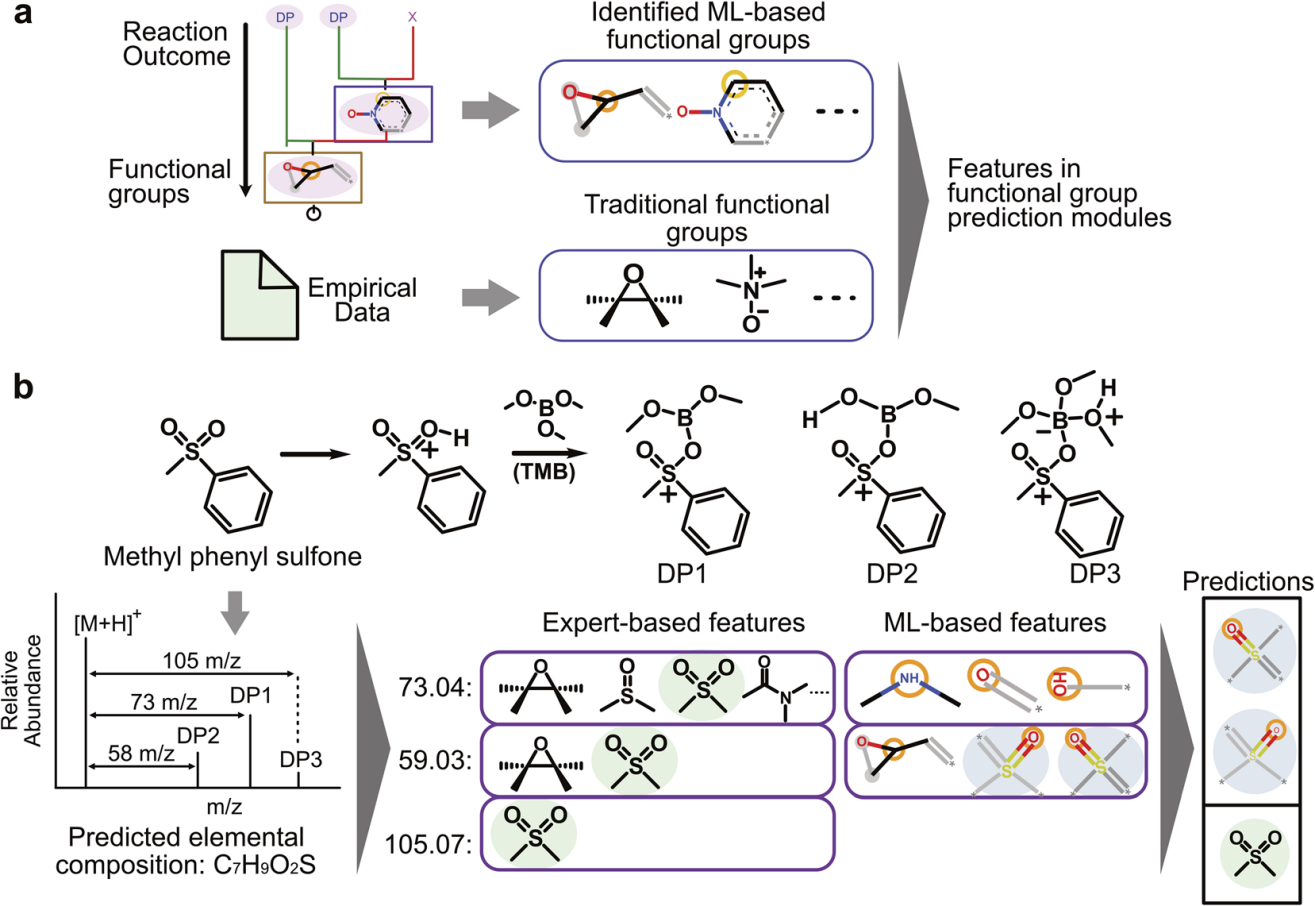
Functional group prediction module. (a) Functional group identification using machine-learning based decision tree models and experimental data. Decision tree models trained with ion–molecule reaction data obtained for each neutral reagent were used to identify functional groups that can form diagnostic products during gas-phase ion–molecule reactions with specific neutral reagents. These identified functional groups were incorporated as features to the functional-group identification module. (b) The module uses an MS^2^ spectrum of the ion–molecule reactions and the measured elemental composition of the unknown analyte as inputs. The output of the module provides functional groups that are likely to exist in the unknown analyte. Diagnostic product ions formed during the reaction are denoted as P1, P2 and P3.

The main objective for the development of the functional-group identification module was to be able to automatically identify functional groups in an unknown protonated analyte given its elemental composition, its gas-phase ion–molecule reaction mass spectrum and other inputs. Fig. 4a summarizes how the ion–molecule reaction data were extracted and used in the module for functional group identification. The reaction outcome (Fig. 4a) denotes whether diagnostic product ions are formed upon reactions with a specific reagent or not. The workflow to identify the functional groups in an unknown analyte is illustrated using the reaction of protonated methyl phenyl sulfone with TMB (Fig. 4b). The MS^2^ mass spectrum measured after the reactions shows three product ions. The mass spectrum is given as an input to the module along with the determined elemental composition, RDBE, and the measured *m*/*z* value of the ionized analyte. The module tests all detected product ions with abundances above a predefined threshold value for the mass differences. Then the module matches all identified mass difference values with the values that should be obtained for the diagnostic product ions formed with the TMB reagent. By jointly considering the elemental composition, the RDBE value, and the mass difference associated with each diagnostic product ion, the model effectively constrains the set of chemically plausible assignments and suppresses overlap between functionalities, thereby minimizing the risk of misassignment.

After such ions (diagnostic ions) have been identified, the module matches them with functional groups that can produce such mass differences by using machine learning and expert-based functional groups. After performing the above steps, the sulfone functional group was predicted as the most probable functional group in the analyte (Fig. 4b).

**Fig. 5:**
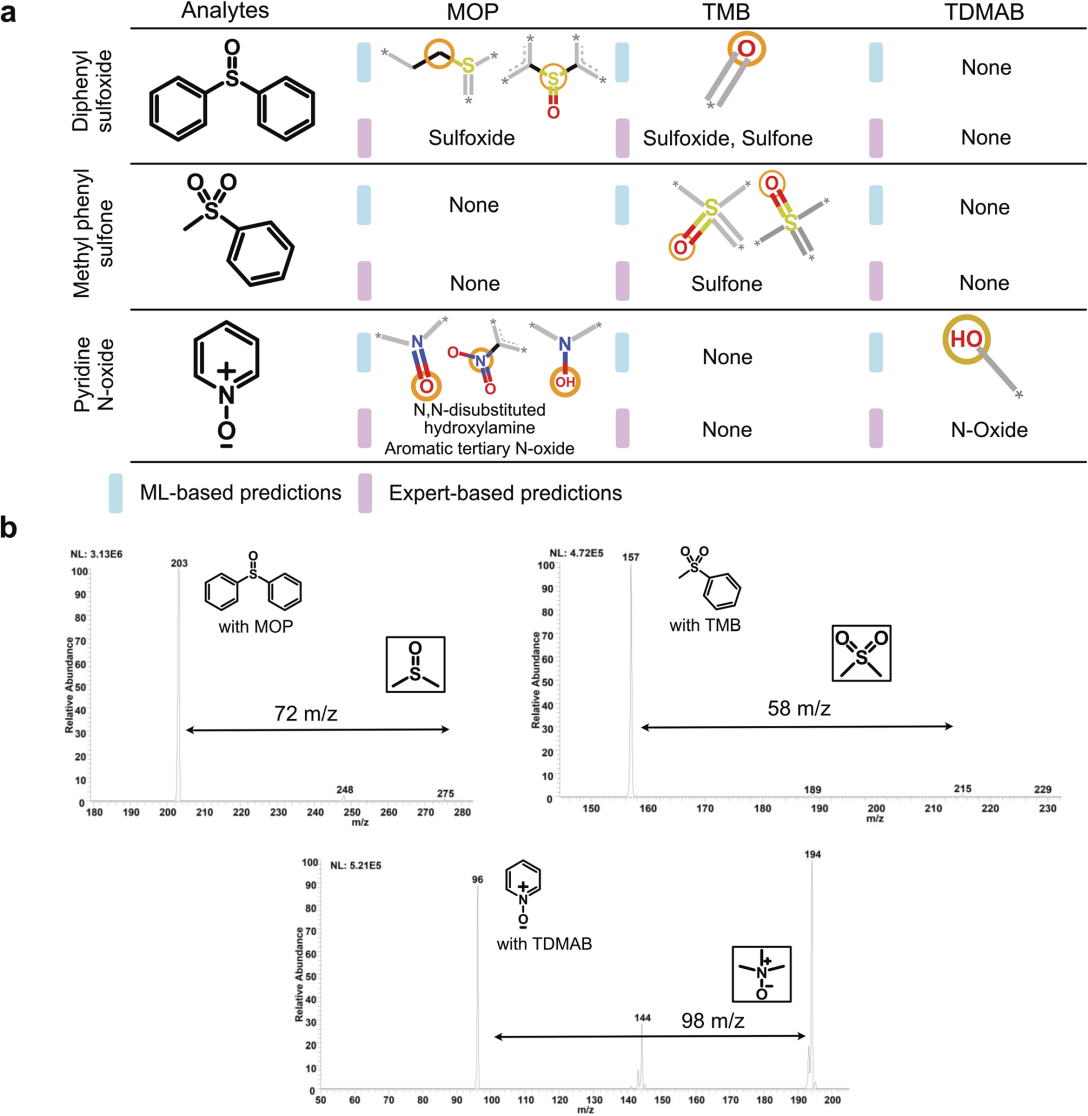
(a) Results obtained for several examples by using the developed functional-group identification module. Each row shows results for a different example and the columns show the neutral reagents and the possible functional groups identified based on the reactions with each neutral reagent. (b) Mass spectra measured after ion–molecule reactions of the protonated analytes with selected reagents and the mass differences that contributed to the selection of the plausible functional groups.

To test the reliability of the module, the reactions of protonated diphenyl sulfoxide, methyl phenyl sulfone, and pyridine *N*-oxide were tested with MOP, TMB and TDMAB reagents. These reactions were not included in the training data set. The module predicted the functional groups shown in Fig. 5a for these analytes. The results are largely in agreement with the actual functional groups of these analytes. A brief summary of these examples is as follows: MOP selectively reacted with the protonated sulfoxide analyte, TMB selectively reacted with the protonated sulfone analyte, and TDMAB selectively reacted with the protonated *N*-oxide analyte. These examples illustrate how the diagnostic product decision trees, the machine-learning identified structural motifs, the expert-defined rules, and the functional-group identification module converge to produce chemically intuitive functional-group assignments. Full, step-by-step decision-path case studies are provided in Section S1.

However, in some cases, such as the reaction of protonated pyridine *N*-oxide with MOP, the module returned multiple plausible functional groups, although the correct assignment remained among the options. The mass-difference values for each analyte are shown in Fig. 5b together with the predicted functional groups. Finally, it should be noted that the module does not return a functional-group prediction when no diagnostic product is formed.

When no diagnostic mass-difference match survives the elemental-composition filter, the platform refrains from suggesting a functional group and flags the analyte as lacking a supported assignment under the current rules.

### 3.3. Performance of decision tree models

As discussed above, machine-learning based decision tree models were developed to identify functional groups based on whether a diagnostic product ion was “formed” or “not formed” during gas-phase ion–molecule reactions. Full decision trees were developed for TMB and TDMAB by using annotated datasets as these decision trees have not been published previously.

To quantify model interpretability, we report model complexity metrics including tree depth (3–5), number of internal nodes (4–12), number of leaves (5–13), and average training-sample support per leaf (1–3). These compact decision trees enable direct mapping of splits to specific Morgan fingerprint bits. Using the decoding procedure described in Section 2.5, we identified the atom environments associated with each major split and grouped them into traditional or nontraditional functional groups. Three representative examples, reactivity of protonated sulfoxides toward TMB, protonated *N*-oxides toward MOP, and protonated tertiary-amines toward TDMAB are presented in Section S1 in the SI. In all cases, the decision paths reflect intuitive chemical reactivity principles.

**Fig. 6:**
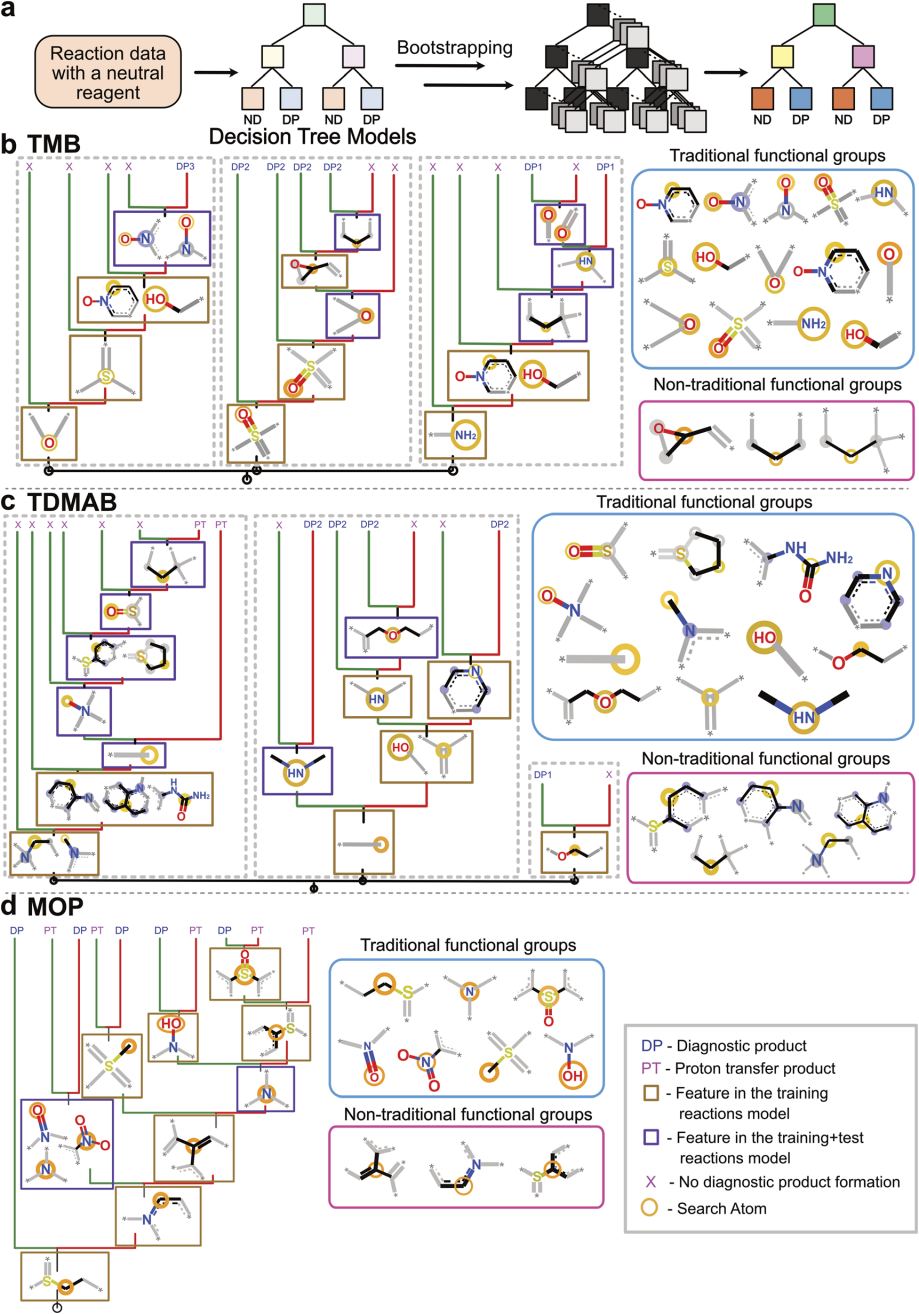
Each dotted box represents a separate decision tree trained for a single diagnostic product (*e.g.*, a specific TMB or TDMAB adduct); the trees are shown together to illustrate the combined reagent-level reactivity, but they are used independently in the functional-group prediction module. (a) Machine-learning decision tree models were trained using ion–molecule reaction data. Bootstrapping was used to check the robustness of the models for different neutral reagents: (b) TMB, (c) TDMAB, and (d) MOP.

The ionic reaction products detected for TMB upon reactions with various protonated analytes were a stable TMB adduct, TMB adduct–MeOH and TMB adduct–Me_2_O. Hence, three decision tree models were developed, one for each of these three reaction pathways (Fig. 6b). Performance of each decision tree was measured using the Cohen's kappa value of the test set. The Cohen's kappa values were 0.74, 0.77, and 0.90 for TMB adduct–MeOH, TMB adduct–Me_2_O, and TMB adduct products, respectively (Cohen's kappa values describe the strength of agreement based on inter-model reliability; values >0.6 indicate a very good or substantial agreement between models and >0.8 indicates an almost perfect agreement). These kappa values correspond to the best-performing combinations of branching-ratio cutoff and Morgan fingerprint radius of the test reactions.

Morgan fingerprint radii were utilized as hyperparameters to fine-tune the developed model. They were varied to optimize the amount of information that should be represented in a chemical structure such as the analyte structure representation as input. On the other hand, measured product ion branching ratios provide the relative abundances (in %) of primary product ions. These values were used to more accurately decide whether a product ion was diagnostic or not. The selected branching ratios and Morgan fingerprint radii are shown in bold in Tables S1–S3 for the three products: TMB adduct–MeOH, TMB adduct–Me_2_O, and TMB adduct. All the data used for preparing the decision trees are shown in Table S6 and the final developed TMB decision tree is a combination of all three developed decision trees for TMB and is shown in Fig. 6b. All test set kappa values obtained indicate an extraordinary ability of the model to classify these diagnostic products, and hence to identify functionalities using diagnostic products.

Similarly, another decision tree model was developed for TDMAB reagent. TDMAB generates two diagnostic product ions upon reactions with certain protonated analytes, which have been previously reported in the literature.^18^ The product ions are an adduct that had eliminated a dimethylamine (DMA) molecule (adduct–DMA), and an adduct that had eliminated two dimethylamine molecules (adduct–2DMA). Two separate decision trees were developed for these two reaction pathways and combined to get the full tree for TDMAB neutral reagent. The Cohen's kappa values obtained were 0.73 and 1.00 for adduct–DMA and adduct–2DMA decision trees respectively. These well-trained decision trees were combined to create the main TDMAB decision tree (Fig. 6a), which was used to identify functionality features that were used to develop the functional-group prediction module.

Although the resulting decision paths are simple and interpretable, they reflect reagent-specific structure–reactivity relationships learned directly from the experimental data, and these patterns extend beyond what could be captured by a manually curated list of traditional functional groups. All reactions used for model training and testing are experimentally determined ion–molecule reactions, drawn from both previously published our own studies and the measurements reported in this work, as listed in Tables S6 and S7. While the external test sets demonstrate generalization across structurally diverse analytes, the current dataset remains limited in size. Expansion to additional analytes and reagents will further strengthen the generalizability of the approach and broaden its applicability.

### 3.4. Reagent prediction module

**Fig. 7:**
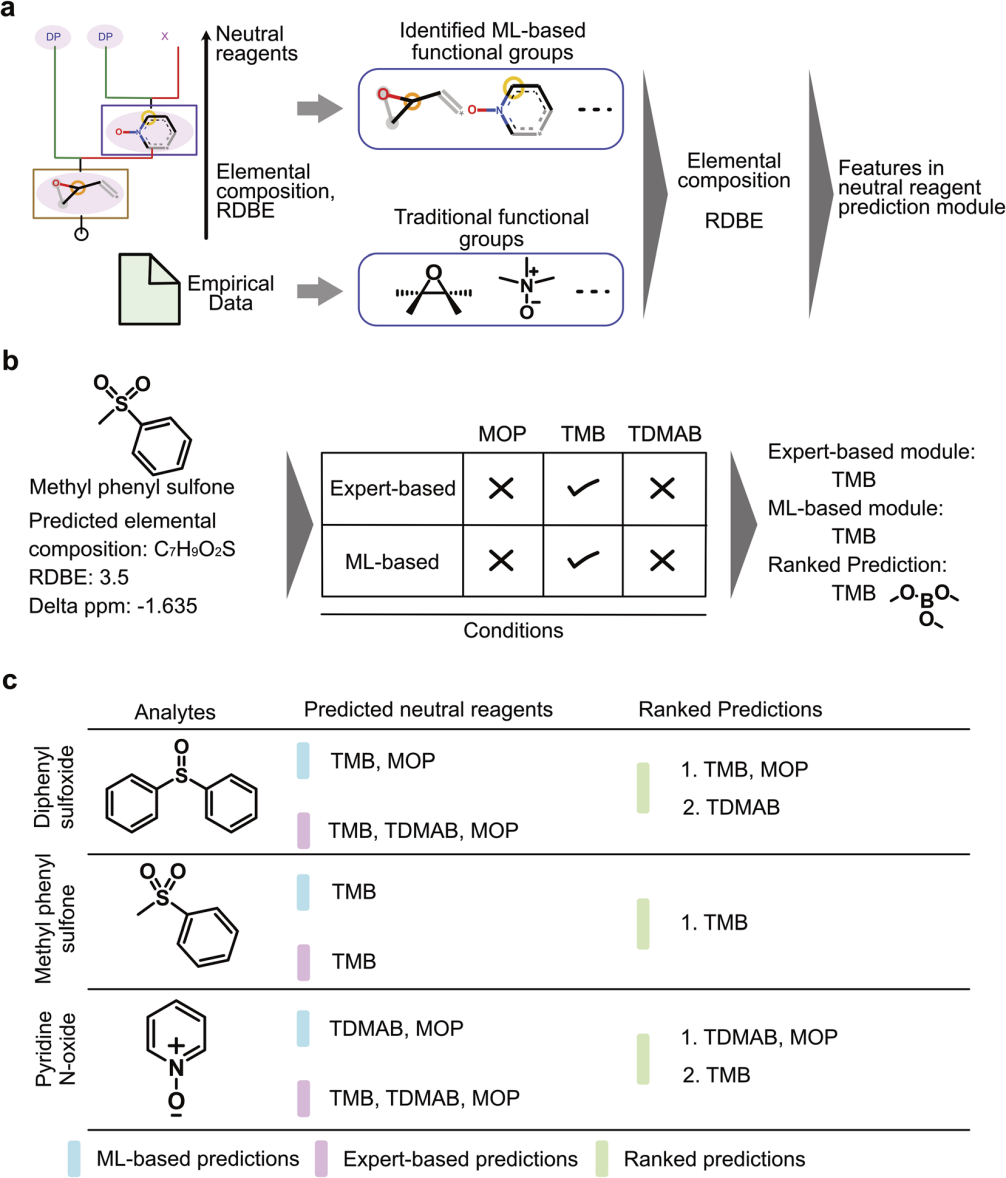
(a) Neutral reagent prediction module: workflow of identifying functional groups by using decision tree models and experimental data. (b) Neutral reagent prediction process for the reaction of protonated methyl phenyl sulfone with the neutral reagent TMB. (c) Neutral reagents predicted for testing the structures of the selected analytes.

A module was developed to predict reagents that are likely to react in an informative manner with an unknown protonated analyte (module workflow shown in Fig. 7a). The functional groups that were identified based on machine learning-based decision tree models were used for the reagent prediction module. The module uses a dataset that contains the measured elemental compositions and RDBE values of known compounds that can form diagnostic products with the reagents considered. These data are matched with the measured elemental compositions and the RDBE values of unknown protonated analytes to make the best prediction of functional groups. If the elemental compositions and RDBE values of the predicted functional groups match the measured elemental composition of the unknown protonated analyte and predicted RDBE values of the functional groups of the unknown analyte, then the module will look up the trained decision tree models to propose reagents that may react with those predicted functional groups in a diagnostic manner. If both machine learning and expert-based approaches suggest the same reagent, it will be prioritized as the top prediction (see Fig. 7b). Finally, all the predictions obtained for reactions of protonated diphenyl sulfoxide, methyl phenyl sulfone, and pyridine *N*-oxide with MOP, TMB and TDMAB reagents (Fig. 7c) are shown as the predicted reagents based on machine learning as well as by expert-based approach. Interestingly, the predicted reagents matched the actual reagents used to carry out diagnostic gas-phase ion–molecule reactions for these testing reactions (Table S11).

### 3.5. Optimization of reagent pulsing-in and pumping-out times

**Fig. 8:**
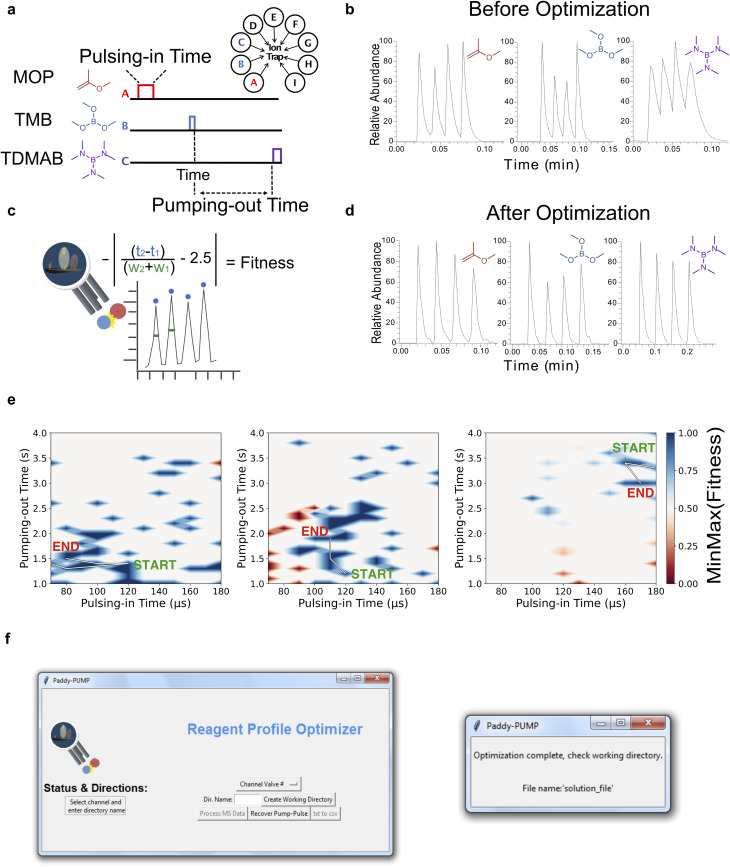
(a) Schematic introducing the pulsing-in and pumping-out times of a pulsing sequence when MOP, TMB, and TDMAB are pulsed into the ion trap. (b) Extracted ion profiles as a function of time are indicative of unoptimized pulsing-in and pumping-out times for MOP, TMB and TDMAB. (c) Depiction of Paddy-PUMP and a schematic displaying the use of resolution within the fitness function being maximized during optimization. Peak width and peak apex are denoted in green and blue text, respectively, in both the equation and extracted ion profile. (d) Extracted ion profiles of the product ions of reactions between protonated methanol dimer and neutral reagent of the optimized pulsing-in and pumping-out time ‘solutions’ produced using Paddy-PUMP. (e) Contour plots displaying the normalized fitness (*via* min–max normalization) of the sampled pulsing-in and pumping-out times, and paths taken in generating solutions. (f) GUI display of Paddy-Pump and pop-up window that informs the experimentor that an optimization experiment is completed.

The reagent pulsing-in time is the time (in µs) that the pulsed valve is opened to allow the reagent to enter the ion trap, while reagent pumping-out time is the time (in seconds) used to pump a reagent out of the ion trap before the next pulsed valve is opened (Fig. 8a). Inadequate pumping-out times of reagents may result in different reagents residing simultaneously in the ion trap, which may complicate the interpretation of the measured data. Previously, the pulsing-in and pumping-out times of the reagents were determined manually. Manual determination is tedious, time-consuming, and inefficient. Paddy-PUMP was utilized to develop automated optimization of the pulsing-in and pumping-out times for MOP, TMB, and TDMAB.

Since the above reagents, MOP, TMB and TDMAB, have been extensively studied, their pulsing-in and pumping-out times that were used previously were first tested by pulsing each reagent into the instrument four times. For MOP, the pulsing-in time used previously^26^ was 150 µs and pumping-out time 1.0 s. The respective extracted ion profile (Fig. 8b) for the product ions (*m*/*z* 73) formed upon reactions of protonated methanol dimer with MOP sightly overlapped near the base of each peak (Fig. 8b, left). On the other hand, TMB was introduced into the ion trap with a pulsing-in time of µs and pumping-out time of 1.2 s, as described in a previous study.^29^ The extracted ion profiles (Fig. 8b) for the ion of *m*/*z* 105 (protonated TMB) formed upon reactions of TMB with protonated methanol dimer also indicated slightly overlapping peaks (Fig. 8b, middle). Lastly, TDMAB was introduced into the ion trap µs and a pumping-out time of 1 s, again according to prior work.^29^ The extracted ion profile (Fig. 8b) for the ion of *m*/*z* 144 (protonated TDMAB) formed upon reactions of TDMAB with protonated methanol dimer had clear and more distinct overlap in its peaks (Fig. 8b, right). The extracted ion profiles (Fig. 8b) show that the 1.0 s pumping-out time for MOP and TDMAB and 1.2 s pumping-out time for TMB were insufficient to fully pump away these reagents, suggesting that these reagents remained in the ion trap and did not completely evacuate from the ion trap before the next pulse.

To ensure that each reagent introduced into the ion trap has been fully removed before the introduction of another reagent, the pulsing-in and the pumping-out times of the reagents were optimized. The selection of peak maxima in extracted ion profiles was facilitated *via* GMM-based heuristics as described above in section 2.8, Optimization of reagent pulsing-in and pumping-out times (Fig. S2). This approach resulted in the successful identification of an optimal pulsing-in schedule for the reagents while excluding noise. Peak apexes were selected to assign a time point (in minutes) to the peak, whereas noise and product ions in the extracted ion profiles with signals below the data dependent thresholds were ignored. An illustrative example of the GMM-based peak method can be found in the SI (Fig. S2a and b). Paddy-PUMP was used to optimize pulsing-in and pumping-out times by utilizing peak pair resolutions in the extracted ion profiles, as mentioned above in section 2.8, Optimization of reagent pulsing-in and pumping-out times (Fig. 8c). The pulsing-in and pumping-out times for MOP were optimized over the course of six iterations (Table S8), with two solutions being generated in the last iteration. The two optimized sets of pulsing-in and pumping-out times were 80 µs and 1.5 s as well as 70 µs and 1.4 s, respectively. The pulsing-in and pumping-out times of TMB were optimized over six iterations (Table S9), with the optimized pulsing-in and pumping-out times being 110 µs and 1.9 s, respectively. Lastly, the pulsing-in and pumping-out times of TDMAB were optimized over three iterations (Table S10), with the optimized pulsing-in and pumping-out times being 170 µs and 3 s, respectively. These optimized values corresponded to shorter pulsing-in times and slightly longer pumping-out times for the selected reagents compared to the previous values.

Optimized pulsing-in and pumping-out times, produced using Paddy-PUMP for all three reagents, display satisfactory resolutions between the peaks in the extracted ion profiles (Fig. 8d). Evolutionary pathways for solutions produced using Paddy-PUMP across the parameter space for each reagent are depicted as contour maps in Fig. 8e. Contour maps displaying the optimization of pulsing-in and pumping-out times with Paddy-PUMP for each individual iteration can be found in the SI (Fig. S6–S8). Lastly, a GUI for Paddy-PUMP was developed to facilitate human-in-the-loop experimentation, with successful identification of sufficient pulsing-in and pumping-out times resulting in a pop-up window being displayed to the experimenter (Fig. 8f). A demo video showcasing the workflow with Paddy-PUMP is provided in the SI (https://doi.org/10.5281/zenodo.17173211).

## 4. Conclusions

An HPLC/APCI MS^2^ LQIT experiment based on diagnostic gas-phase ion–molecule reactions and introduction of reagents *via* a set of nine pulsed valves were coupled with bootstrapped human interpretable machine learning models to automate the identification of functionalities in protonated analytes in mixtures. Furthermore, an automated system was developed to optimize the reagent pulsing-in and pumping-out times for the sequential introduction of several reagents by use of an in-house evolutionary algorithm.

A decision tree developed^36^ previously for MOP was utilized in this research, together with new decision trees developed for TDMAB and TMB. Bootstrapping techniques were used to select reagent-specific decision trees from 10 000 models and the performance was assessed using kappa statistics to select the best performing model (kappa > 0.7 indicates good inter-model reliability compared to the model prediction by chance). The identification of probable functionalities in the unknown protonated analytes is based on diagnostic product ions formed upon ion–molecule reactions as well as the ring and double-bond equivalents and elemental compositions obtained from high-resolution accurate mass measurements for the protonated analyte. The diagnostic ion–molecule reaction products were identified based on the *m*/*z* differences between the protonated analytes and the detected product ions. The machine learning models trained on known diagnostic ion–molecule reactions identified traditional (expert-based) and nontraditional (graph-based) functional group features. Development of a method for the selection of suitable reagents for previously unstudied analytes was based on a machine learning model that utilized the measured elemental composition of the protonated analyte and its ring and double bond equivalent as well as the decision trees developed for the neutral reagents.

The work presented here demonstrates that the combination of machine learning and tandem mass spectrometry based on diagnostic gas-phase ion–molecule reactions can be used to rapidly identify functional groups in unknown protonated compounds directly in mixtures. Incorporating machine learning with ion–molecule reactions will facilitate the interpretation of the data and selection of reagents for previously unstudied analytes. Because the machine learning workflow is reagent-agnostic, the same training, validation, and functional-group extraction procedures can be applied to historic and newly studied reagents, allowing this platform to be systematically expanded as additional ion–molecule reaction data become available. The Paddy-PUMP method was used to optimize the reagent pulsing-in and pumping-out times. The optimal reagent conditions for MOP (pulsing-in time: 80 µs, pumping-out time 1.5 s; or pulsing-in time: 70 µs, pumping-out time: 1.4 s), TMB (pulsing-in time: 110 µs, pumping-out time 1.9 s) and TDMAB (pulsing-in time: 170 µs, pumping-out time: 3.0 s) were determined. These optimized values provided shorter pulsing-in times and slightly longer pumping-out times compared to the previously used values. Computer codes, scripts and additional information can be found in GitHub (https://github.com/chopralab/cbm_ml_automation) and in the SI.

Demo (SI Movie: PADDY_PUMP_DEMO.mp4), https://doi.org/10.5281/zenodo.17173211. The demo shows the process from designing to performing experiments for the optimization of pulsing-in and pumping-out times of reagents by use of the Paddy-PUMP app.

## Author contributions

Armen G. Beck: writing – review & editing, writing – original draft, visualization, validation, software, methodology, investigation, formal analysis, data curation, conceptualization. Ruth O. Anyaeche: writing – review & editing, writing – original draft, visualization, validation, methodology, investigation, formal analysis, data curation, conceptualization. Prageeth Wijewardhane: writing – review & editing, writing – original draft, visualization, validation, software, methodology, investigation, formal analysis, data curation, conceptualization. Sanjay Iyer: software, methodology, investigation. Yue Fu: visualization, methodology. Judy Kuan-Yu Liu: visualization, methodology, conceptualization. Jifa Zhang: methodology, conceptualization. Kawthar Z. Alzarieni: visualization, methodology, conceptualization. Erlu Feng: methodology. Ryan T. Hilger: software, methodology. Christopher Welch: resources. Hilkka I. Kenttämaa: writing – review & editing, supervision, methodology, resources, funding acquisition, conceptualization. Gaurav Chopra: writing – review & editing, supervision, methodology, resources, funding acquisition, conceptualization, project administration.

## Conflicts of interest

The authors declare the following competing financial interest(s): G. C. is the Director of the Merck-Purdue Center funded by Merck Sharp & Dohme, a subsidiary of Merck and the co-founder of Meditati Inc., BrainGnosis Inc. and LIPOS BIO Inc. The remaining authors declare no competing interests.

## Supplementary Material

SC-OLF-D5SC07324C-s001

## Data Availability

All data and computer code related to the manuscript is available at GitHub at https://github.com/chopralab/cbm_ml_automation. Supplementary information (SI): branching ratio cutoffs, reactions used for training decision trees, Paddy generated pumping-in and pumping-out parameters and results, and case studies for chemical interpretation of models. See DOI: https://doi.org/10.1039/d5sc07324c.
