# Supplementary material for: Interpretable machine learning-based automated HPLC/MS2 platform using ion–molecule reactions for the identification of functionalities in analytes
Source: Chem Sci. 2026 Jun 2. Online ahead of print. doi: 10.1039/d5sc07324c (PMC13322741; doi:10.1039/d5sc07324c)
Supplement: SC-OLF-D5SC07324C-s001 [file SC-OLF-D5SC07324C-s001.pdf]

Supplementary Information (SI) for Chemical Science.
This journal is © The Royal Society of Chemistry 2026

Supporting Information

Interpretable Machine Learning-based Automated HPLC/MS^2 Platform using Ion-Molecule Reactions for the Identification of Functionalities in Analytes

Armen G. Beck,[a,+] Ruth O. Anyaeche,[a,+] Prageeth Wijewardhane,[a,+] Sanjay Iyer,[a] Yue Fu,[a] Judy Kuan-Yu Liu,[a] Jifa Zhang,[a] Kawthar Z. Alzarieni,[b] Erlu Feng,[a] Ryan T. Hilger,[a] Christopher Welch,[d] Hilkka I. Kenttämaa,[a,*] Gaurav Chopra[ac,*]

[a] Department of Chemistry, Purdue University, 560 Oval Drive, West Lafayette, IN, USA.

[b] Department of Medicinal Chemistry and Pharmacognosy, Faculty of Pharmacy, Jordan University of Science and Technology, P.O. Box 3030, Ar-Ramtha Street, Irbid 22110, Jordan

[c] Department of Computer Science (*by courtesy*), Purdue Institute for Drug Discovery, Regenstrief Center for Healthcare Engineering, Purdue Center for Cancer Research, Purdue Institute for Inflammation, Immunology and Infectious Disease, Purdue Institute for Integrative Neuroscience, West Lafayette, IN 47909 USA

[d] Indiana Consortium for Analytical Science & Engineering (ICASE), Indianapolis, Indiana 46202, USA

+These authors contributed equally to this work

* E-mail: hilkka@purdue.edu; gchopra@purdue.edu

Table of Contents

Table S1. Additional branching ratio cutoffs and Morgan fingerprint radii for the **TMB adduct - MeOH** decision tree model. 4

Table S2. Additional branching ratio cutoffs and Morgan fingerprint radii for the **TMB adduct – Me_2O** decision tree model. 5

Table S3. Additional branching ratio cutoffs and Morgan fingerprint radii for the **TMB adduct** decision tree model. 6

Table S4. Additional branching ratio cutoffs and Morgan fingerprint radii for the **TDMAB adduct - dimethylamine** decision tree model. 7

Table S5. Additional branching ratio cutoffs and Morgan fingerprint radii for the **TDMAB adduct – 2 dimethylamine** decision tree model. 8

Table S6. Reactions used to train decision tree models with TMB neutral reagent. Diagnostic product branching ratios are shown for adduct-methanol, adduct, and adduct-dimethyl oxide products, respectively. Structures of formed diagnostic product species are shown right of reaction arrows, with dashed lines indicating no diagnostic product of a given species formed. 9

Table S7. Reactions used to train decision tree models with TDMAB neutral reagent. Diagnostic product branching ratios are shown for adduct, adduct-dimethylamine, and adduct-2 dimethylamine products, respectively. Structures of formed diagnostic product species are shown right of reaction arrows, with dashed lines indicating no diagnostic product of a given species formed. 14

Table S8. Parameters generated by Paddy when optimizing introduction of MOP and its resolution values. 19

Table S9. Parameters generated by Paddy when optimizing introduction of TMB and its resolution values. 22

Table S10. Parameters generated by Paddy when optimizing introduction of TDMAB and its resolution values. 25

Table S11. Experimentally identified neutral reagents that can undergo diagnostic ion-molecule reactions with the protonated analytes shown below. Machine learning based predictions are shown in the last column. 26

Table S12. Test set diversity of each neutral reagent model 26

Figure S1. Truncated extracted ion profile as a function of experiment time for the zeroth (initiation) iteration of the optimization of pulsing-in and pumping-out times for TDMAB. Displayed are the initial five tuning peaks and two subsequent Paddy generated peak clusters. The blue dots depict the identified times for peak maxima and green lines as the width of the peaks at the half height. Black vertical lines display the anticipated midpoints between peak clusters calculated using the pumping-out times from the recipe file. Midpoints are used to segment extracted ion profiles such to allow association of programed pulsing-in of reagents to their resulting fitness/resolution. 27

Figure S2. Overview of data dependent peak selection via Gaussian mixture models (GMMs), with the extracted ion profile being the fifth iteration of the optimization of pulsing-in and pumping-out times for TMB. **a**) Extracted ion profile as a function of experiment time with peaks identified by the SciPy 'find_peaks' function indicated by orange dots while using an arbitrary peak height threshold of 300. **b**) Extracted ion profile as a function of experiment time with peaks identified based on the GMM based threshold. Noise peaks and peaks programed by Paddy-PUMP with insufficient height have not been selected for time domain orientation or resolution calculations. **c**) Datapoints selected from the TBM

extracted ion profile and sorted from high to low **value**, and clustered using a GMM. Clusters are colored separately, with the GMM intensity threshold defined in the methods section in main text (**eq. 3**)...........27

Figure S6. Contour maps displaying MOP optimization with Paddy-PUMP over six iterations. Evaluated parameters (Paddy plants) involved in yielding the solutions for the experiment are displayed as green dots with their seed number (Table S8) displayed beside them. Plants from prior iterations are displayed as a darker hue of green, and an arrow points from them to their progeny (new parameters generated via sampling a distribution centered on the prior parameters). If a plant does not produce a seed directly involved in generating one of the optimized sets of pulsing-in and pumping-out times during an iteration it remains bright green with no arrow until the iteration in which it does...29

Figure S7. Contour maps displaying TMB optimization with Paddy-Pump over six iterations. Fitness values are min-max normalized to range from zero to one and displayed using a divergent blue-red color scale. Evaluated parameters (Paddy plants) involved in yielding the solution for the experiment are displayed as green dots with their seed number (Table S9) displayed beside them. Plants from prior iterations are displayed as a darker hue of green, and an arrow points from them to their progeny (new parameters generated via sampling a distribution centered on the prior parameters). If a plant does not produce a seed directly involved in generating the optimized set of pulsing-in and pumping-out times during an iteration it remains bright green with no arrow until the iteration it does. 30

Figure S8. Contour maps displaying TDMAB optimization with Paddy-Pump over three iterations. Fitness values are min-max normalized to range from zero to one, and displayed using a divergent color blue-red scale. Evaluated parameters (Paddy plants) involved in yielding the solution for the experiment are displayed as green dots with their seed number (Table S10) displayed beside them. Plants from prior iterations are displayed as a darker hue of green, and an arrow points from them to their progeny (new parameters generated via sampling a distribution centered on the prior parameters). 30

Figure S9. a) Mass spectra showing a selective ion-molecule reaction between protonated pyridine N-oxide and TDMAB, alongside spectra demonstrating no selective reaction with MOP or TMB. b) Mass spectra illustrating a selective ion-molecule reaction between protonated methyl phenyl sulfone and TMB, with no reaction observed for protonated methyl phenyl sulfone with TDMAB or MOP. 31

Section S1. Three Case Studies Linking Decision Paths to Chemical Rationale 31

Tables S1–S5 summarize the validation of the decision tree models for all reagents and diagnostic product channels. For each reagent, a fixed held-out test set of five reactions was used to assess generalization performance, while the remaining reactions constituted the training set. For every combination of branching-ratio cutoff and Morgan fingerprint radius, model reliability on the training data was quantified using leave-one-out cross-validation (LOOCV), and the corresponding F1 score, False detection rate (FDR), and Kappa values are reported in rows labeled "F1 Score", "FDR", and "Kappa". Predictive consistency on the five held-out test reactions was evaluated by training an ensemble of 10,000 decision trees and reporting, for each compound, the fraction of correct predictions across the ensemble. These per-compound fractions are shown in the rows labeled "Compound 1–5."

Table S1. Additional branching ratio cutoffs and Morgan fingerprint radii for the **TMB adduct - MeOH** decision tree model.

Compound	Radius	Branching Ratio Cutoff							
		0.2	0.3	0.4	0.5	0.6	0.7	0.8	0.9
1	0	0.67	0.67	0.67	0.67	0.67	1.00	1.00	0.00
2		0.67	0.67	0.67	0.67	0.67	1.00	1.00	1.00
3		1.00	1.00	1.00	1.00	1.00	0.95	1.00	1.00
4		1.00	0.67	0.67	0.67	0.67	0.67	0.67	0.00
5		1.00	1.00	1.00	1.00	1.00	0.69	0.00	0.00
F1		0.94	0.83	0.83	0.83	0.83	0.69	0.80	0.80
FDR		0.18	0.25	0.25	0.25	0.25	0.42	0.50	0.40
Kappa		0.74	0.44	0.44	0.44	0.44	0.19	0.61	0.74
1	1	0.67	0.67	0.67	0.67	0.67	1.00	1.00	0.00
2		0.67	0.67	0.67	0.67	0.67	1.00	1.00	1.00
3		1.00	1.00	1.00	1.00	1.00	0.94	1.00	1.00
4		1.00	0.67	0.67	0.67	0.67	0.67	0.61	0.00
5		1.00	1.00	1.00	1.00	1.00	0.58	0.00	0.00
F1		0.91	0.83	0.83	0.83	0.83	0.69	0.67	0.80
FDR		0.22	0.29	0.29	0.29	0.29	0.46	0.67	0.50
Kappa		0.59	0.44	0.44	0.44	0.44	0.19	0.33	0.74
1	**2**	1.00	1.00	1.00	1.00	1.00	1.00	1.00	0.00
2		1.00	1.00	1.00	1.00	1.00	1.00	1.00	1.00
3		0.50	1.00	1.00	1.00	1.00	0.67	1.00	1.00
4		1.00	0.33	0.33	0.33	0.33	0.33	0.50	0.00
5		1.00	1.00	1.00	1.00	1.00	0.67	0.00	0.00
F1		0.94	0.83	0.83	0.83	0.83	0.83	0.67	0.80
FDR		0.22	0.29	0.29	0.29	0.29	0.40	0.70	0.50
Kappa		**0.74**	0.44	0.44	0.44	0.44	0.46	0.33	0.74

Table S2. Additional branching ratio cutoffs and Morgan fingerprint radii for the **TMB adduct – Me_2O** decision tree model.

Compound	Radius	Branching Ratio Cutoff														
		0	0.01	0.02	0.03	0.04	0.05	0.06	0.07	0.08	0.09	0.1	0.11	0.12	0.13	0.14
1	0	1.00	1.00	1.00	1.00	1.00	1.00	1.00	1.00	0.50	0.00	0.00	0.00	0.00	0.00	1.00
2		0.67	0.67	0.67	0.00	0.00	0.00	0.00	0.00	0.00	0.00	0.00	0.00	0.00	0.00	1.00
3		1.00	1.00	1.00	0.50	0.50	0.50	0.50	0.25	0.25	0.33	0.00	0.00	0.00	0.00	1.00
4		0.00	0.00	0.00	0.00	0.00	0.00	0.00	0.00	0.00	0.00	0.33	0.33	0.00	0.00	1.00
5		0.67	0.67	0.67	0.00	0.00	0.00	0.00	0.00	0.00	0.00	0.00	0.00	0.00	0.00	1.00
F1		0.62	0.62	0.62	0.58	0.58	0.58	0.58	0.79	0.53	0.74	n.d.	n.d.	n.d.	n.d.	n.d.
FDR		0.80	0.80	0.80	0.71	0.71	0.71	0.71	0.86	0.67	0.80	0.00	0.00	0.00	0.00	0.00
Kappa		0.69	0.69	0.69	0.55	0.55	0.55	0.55	0.77	0.51	0.52	-0.16	-0.16	-0.06	-0.06	n.d.
1	**1**	1.00	1.00	1.00	1.00	1.00	1.00	1.00	1.00	0.50	0.00	0.11	0.11	0.00	0.00	1.00
2		0.67	0.67	0.67	0.00	0.00	0.00	0.00	0.00	0.00	0.00	0.11	0.11	0.00	0.00	1.00
3		1.00	1.00	1.00	0.50	0.50	0.50	0.50	0.00	0.00	0.00	0.00	0.00	0.00	0.00	1.00
4		0.00	0.00	0.00	0.00	0.00	0.00	0.00	0.00	0.00	0.00	0.33	0.33	0.00	0.00	1.00
5		0.67	0.67	0.67	0.00	0.00	0.00	0.00	0.00	0.00	0.00	0.11	0.11	0.00	0.00	1.00
F1		0.74	0.74	0.74	0.71	0.71	0.71	0.71	0.86	0.77	0.50	0.00	0.00	0.00	0.00	0.00
FDR		0.40	0.40	0.40	0.38	0.43	0.43	0.43	0.20	0.50	1.00	n.d.	n.d.	n.d.	n.d.	n.d.
Kappa		0.58	0.58	0.58	0.43	0.43	0.43	0.43	**0.77**	0.51	0.19	0.00	0.00	0.00	0.00	n.d.
1	2	1.00	1.00	1.00	1.00	1.00	1.00	1.00	1.00	0.50	0.00	0.20	0.20	0.00	0.00	1.00
2		0.67	0.67	0.67	0.00	0.00	0.00	0.00	0.00	0.00	0.00	0.20	0.20	0.00	0.00	1.00
3		1.00	1.00	1.00	0.50	0.50	0.50	0.50	0.00	0.00	0.00	0.00	0.00	0.00	0.00	1.00
4		0.00	0.00	0.00	0.00	0.00	0.00	0.00	0.00	0.00	0.00	0.33	0.33	0.00	0.00	1.00
5		0.67	0.67	0.67	0.00	0.00	0.00	0.00	0.00	0.00	0.00	0.13	0.13	0.00	0.00	1.00
F1		0.74	0.74	0.74	0.67	0.67	0.67	0.67	0.80	0.71	0.40	0.00	0.00	0.00	0.00	0.00
FDR		0.45	0.45	0.45	0.43	0.43	0.43	0.43	0.43	0.67	1.00	n.d.	n.d.	n.d.	n.d.	n.d.
Kappa		0.58	0.58	0.58	0.33	0.33	0.33	0.33	0.67	0.42	0.11	0.00	0.00	0.00	0.00	n.d.

n.d. denotes not defined or not a number

Table S3. Additional branching ratio cutoffs and Morgan fingerprint radii for the **TMB adduct** decision tree model.

Compound	Radius	Branching Ratio Cutoff										
		0	0.04	0.08	0.12	0.16	0.2	0.24	0.28	0.32	0.36	0.4
1	0	1.000	0.333	0.000	0.000	0.000	0.000	0.000	0.000	0.000	0.000	0.000
2		1.000	0.333	0.000	0.000	0.000	0.000	0.000	0.000	0.000	0.000	0.000
3		0.000	0.000	0.000	0.000	0.000	0.000	0.000	0.000	0.000	0.000	0.000
4		1.000	1.000	1.000	1.000	0.667	0.667	0.667	0.667	0.667	0.667	0.667
5		1.000	0.333	0.500	0.500	0.500	0.500	0.500	0.500	0.500	0.000	0.000
F1		0.880	0.750	0.800	0.800	0.714	0.714	0.714	0.714	0.727	0.667	0.667
FDR		0.182	0.500	0.167	0.167	0.400	0.400	0.400	0.400	0.400	0.500	0.500
Kappa		0.704	0.438	0.690	0.690	0.571	0.571	0.571	0.571	0.632	0.577	0.577
1	1	1.000	0.333	0.000	0.000	0.000	0.000	0.000	0.000	0.000	0.000	0.000
2		1.000	0.333	0.000	0.000	0.000	0.000	0.000	0.000	0.000	0.000	0.000
3		0.000	0.000	0.333	0.333	0.333	0.333	0.333	0.333	0.500	0.000	0.000
4		1.000	1.000	1.000	1.000	0.667	0.667	0.667	0.667	0.166	0.667	0.667
5		1.000	0.333	0.333	0.333	0.333	0.333	0.333	0.333	0.500	0.000	0.000
F1		0.923	0.750	0.800	0.800	0.615	0.615	0.714	0.615	0.545	0.667	0.667
FDR		0.231	0.636	0.250	0.250	0.667	0.667	0.667	0.667	0.833	0.600	0.600
Kappa		0.800	0.341	0.690	0.690	0.571	0.571	0.444	0.571	0.386	0.577	0.577
1	**2**	1.000	0.500	0.000	0.000	0.000	0.000	0.000	0.000	0.000	0.000	0.000
2		1.000	0.500	0.000	0.000	0.000	0.000	0.000	0.000	0.000	0.000	0.000
3		0.000	0.667	0.333	0.333	0.333	0.333	0.333	0.333	0.500	0.500	0.500
4		1.000	1.000	1.000	1.000	0.667	0.667	0.667	0.667	0.000	0.000	0.000
5		1.000	0.667	0.333	0.333	0.333	0.333	0.333	0.333	0.500	0.000	0.000
F1		0.960	0.667	0.923	0.923	0.600	0.600	0.600	0.600	0.600	0.857	0.857
FDR		0.231	0.545	0.000	0.000	0.600	0.600	0.600	0.600	0.500	0.333	0.333
Kappa		**0.901**	0.417	0.889	0.889	0.481	0.481	0.481	0.481	0.475	0.829	0.829

Table S4. Additional branching ratio cutoffs and Morgan fingerprint radii for the **TDMAB adduct - dimethylamine** decision tree model.

Compound	Radius	Branching Ratio Cutoff										
		0.00	0.10	0.20	0.30	0.40	0.50	0.60	0.70	0.80	0.90	1
1	0	1.00	0.25	0.25	0.25	0.08	0.00	0.00	0.00	0.00	0.00	1
2		1.00	1.00	1.00	1.00	1.00	1.00	1.00	0.21	0.21	0.33	1
3		1.00	1.00	1.00	1.00	0.83	0.24	0.50	0.37	0.37	0.33	1
4		0.67	1.00	1.00	0.42	0.50	0.24	0.13	0.10	0.11	0.67	1
5		1.00	1.00	1.00	1.00	0.53	0.24	0.55	0.21	0.21	0.67	1
F1		0.98	0.93	0.93	0.84	0.65	0.74	0.73	0.38	0.38	0.36	0.00
FDR		0.04	0.13	0.13	0.26	0.50	0.50	0.58	0.89	0.89	0.75	n.d.
Kappa		0.00	0.73	0.73	0.54	0.25	0.52	0.43	0.14	0.14	0.23	n.d.
1	1	1.00	0.00	0.00	0.00	0.00	0.00	0.00	0.00	0.00	0.00	1
2		1.00	1.00	1.00	1.00	1.00	1.00	1.00	0.00	0.00	0.00	1
3		1.00	1.00	1.00	0.73	1.00	0.51	0.00	0.67	0.67	0.00	1
4		1.00	1.00	1.00	0.67	1.00	0.75	0.50	0.67	0.67	0.70	1
5		1.00	1.00	1.00	1.00	1.00	1.00	1.00	0.67	0.67	0.70	1
F1		0.96	0.89	0.93	0.73	0.59	0.81	0.80	0.33	0.43	0.36	0.00
FDR		0.04	0.15	0.15	0.41	0.54	0.50	0.50	0.89	0.89	1.00	n.d.
Kappa		-0.04	0.59	0.66	0.03	0.11	0.59	0.65	0.12	0.12	0.23	n.d.
1	**2**	1.00	0.00	0.00	0.05	0.28	0.00	0.00	0.00	0.00	0.00	1
2		1.00	1.00	1.00	1.00	1.00	1.00	1.00	0.33	0.34	0.00	1
3		1.00	1.00	1.00	0.62	1.00	0.50	0.00	0.16	0.17	0.00	1
4		1.00	1.00	1.00	0.67	1.00	0.88	0.43	0.00	0.00	0.00	1
5		1.00	1.00	1.00	1.00	1.00	1.00	0.83	0.00	0.00	0.00	1
F1		0.98	0.91	0.89	0.74	0.56	0.81	0.80	0.18	0.18	0.50	0.00
FDR		0.04	0.15	0.15	0.41	0.57	0.43	0.50	1.00	1.00	1.00	n.d.
Kappa		0.00	**0.73**	0.66	-0.03	-0.03	0.65	0.65	0.04	0.04	0.37	n.d.

n.d. denotes not defined or not a number

Table S5. Additional branching ratio cutoffs and Morgan fingerprint radii for the **TDMAB adduct – 2 dimethylamine** decision tree model.

Compound	Radius	Branching Ratio Cutoff							
		0.000	0.050	0.100	0.150	0.200	0.300	0.500	1.000
1	0	1.000	1.000	1.000	0.000	0.000	0.000	0.000	1.000
2		0.000	0.000	0.000	0.000	0.000	0.000	0.000	1.000
3		1.000	1.000	1.000	0.000	0.000	0.000	0.000	1.000
4		0.000	0.000	0.000	0.000	0.000	0.000	0.000	1.000
5		0.000	0.000	0.000	0.000	0.000	0.000	0.000	1.000
F1		0.500	0.570	0.570	0.67	0.67	0.67	0	0
FDR		0.33	0.33	0.33	n.d.	n.d.	n.d.	n.d.	n.d.
Kappa		0.294	0.634	0.634	0	0	0	0	n.d.
1	1	0.788	0.782	0.784	1.000	1.000	1.000	0.500	1.000
2		0.000	0.000	0.000	0.000	0.000	0.000	0.000	1.000
3		0.786	0.788	0.792	0.000	0.000	0.000	0.000	1.000
4		0.000	0.000	0.000	0.000	0.000	0.000	0.000	1.000
5		0.000	0.000	0.000	0.000	0.000	0.000	0.000	1.000
F1		0.29	0.57	0.57	1.00	1.00	1.00	0.00	0.00
FDR		0.80	0.60	0.60	n.d.	n.d.	n.d.	n.d.	n.d.
Kappa		0.211	0.516	0.516	1.000	1.000	1.000	0.000	n.d.
1	**2**	0.688	0.689	0.684	0.331	0.331	0.335	0.285	1.000
2		0.000	0.000	0.000	0.000	0.000	0.000	0.000	1.000
3		0.767	0.768	0.770	0.000	0.000	0.000	0.000	1.000
4		0.000	0.000	0.000	0.000	0.000	0.000	0.000	1.000
5		0.000	0.000	0.000	0.000	0.000	0.000	0.000	1.000
F1		0.286	0.571	0.571	1.000	1.000	1.000	0.000	0.000
FDR		1.000	0.667	0.667	n.d.	n.d.	n.d.	n.d.	n.d.
Kappa		0.211	0.516	0.516	**1.000**	1.000	1.000	0.000	n.d.

n.d. denotes not defined or not a number

Table S6. Reactions used to train decision tree models with TMB neutral reagent. Diagnostic product branching ratios are shown for adduct-methanol, adduct, and adduct-dimethyl oxide products, respectively. Structures of formed diagnostic product species are shown right of reaction arrows, with dashed lines indicating no diagnostic product of a given species formed.

Analyte number	Training set reactions	Diagnostic product branching ratio
1	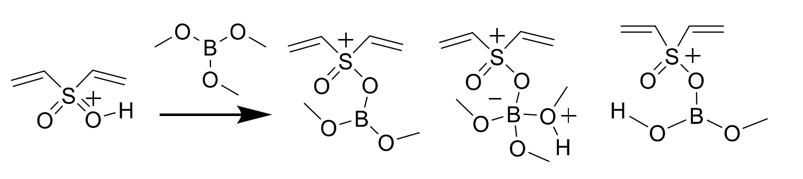	0.85, 0.05, 0.1
2	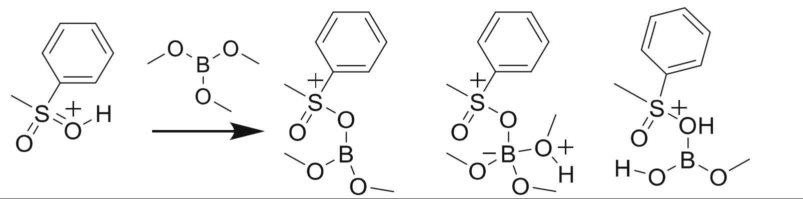	0.87, 0.03, 0.1
3	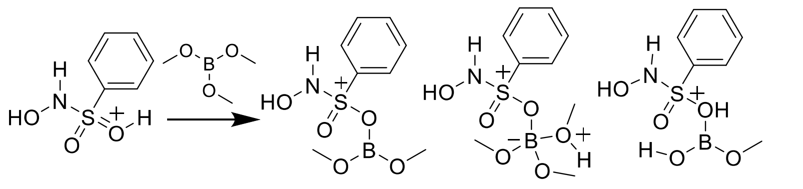	0.74, 0.14, 0.12
4	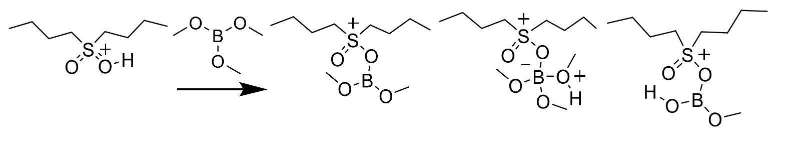	0.89, 0.01, 0.09
5	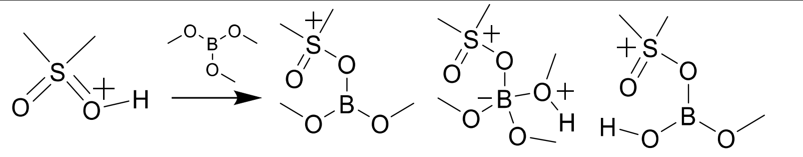	0.83, 0.03, 0.08
6	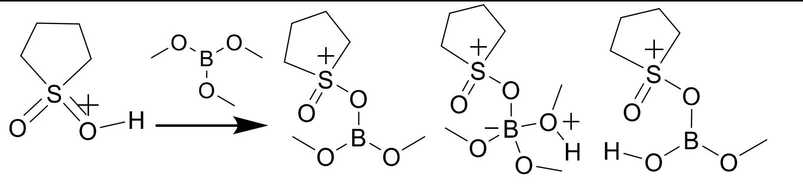	0.79, 0.07, 0.14
7	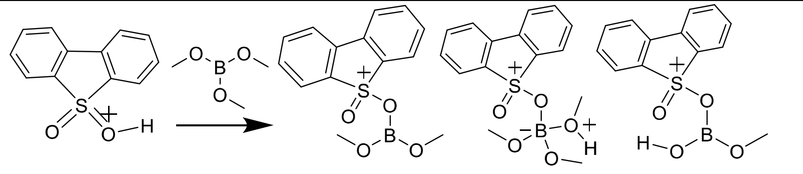	0.89, 0.01, 0.1

8	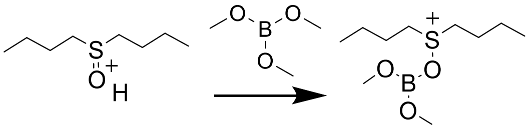	1, 0, 0
9	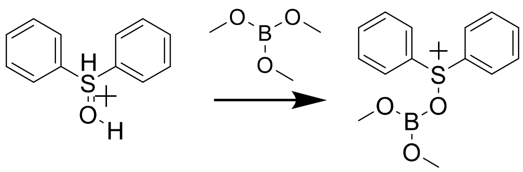	1, 0, 0
10	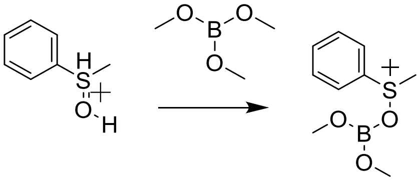 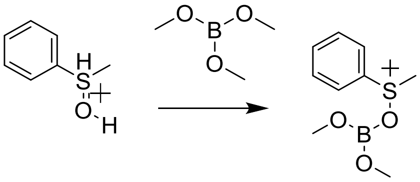 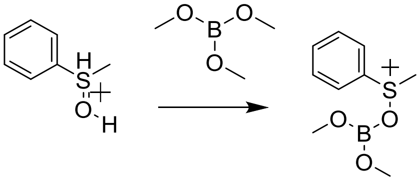 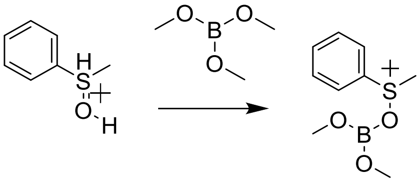 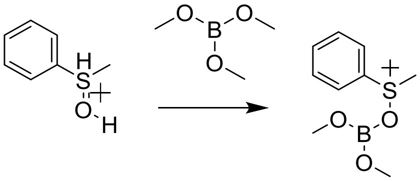 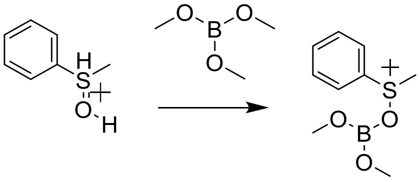	1, 0, 0
11	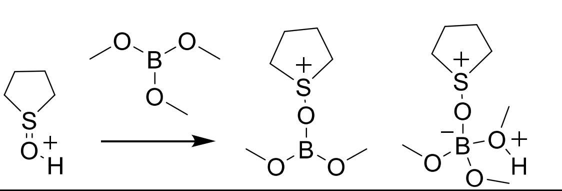	0.64, 0.36, 0

12	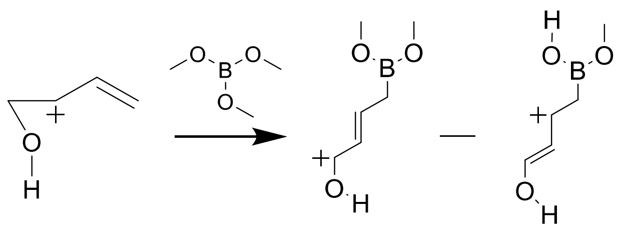	0.9, 0, 0.1
13	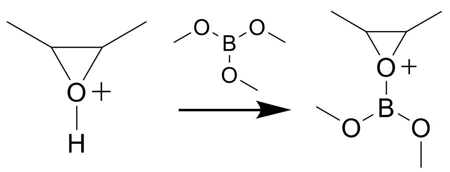	1, 0, 0
14	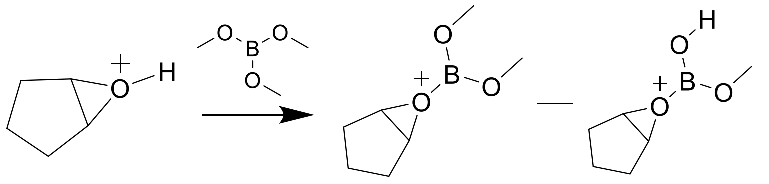	0.97, 0, 0.03
15	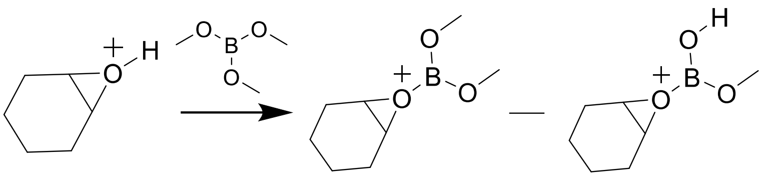	0.9, 0, 0.1
16	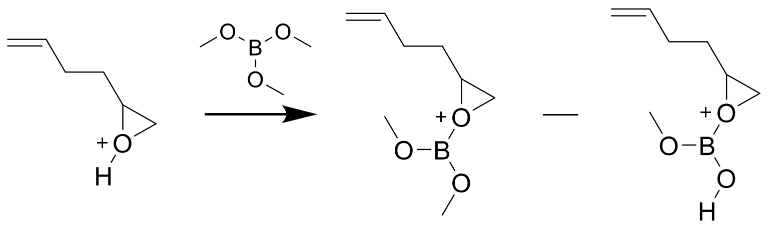	0.93, 0, 0.07
17	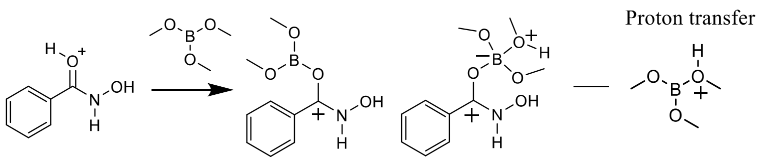	0.26, 0.29, 0, 0.45*
18	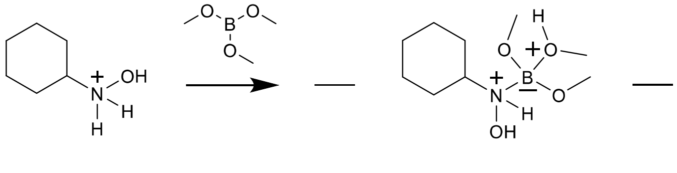	0, 1, 0

19	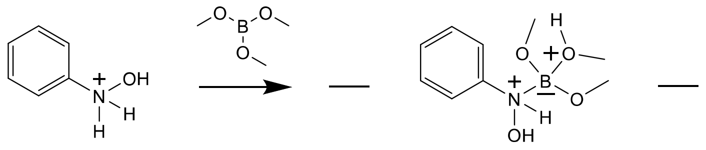	0, 1, 0
20	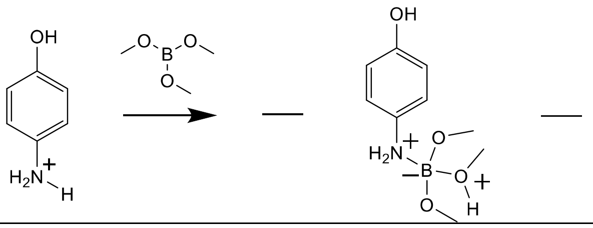	0, 1, 0
21	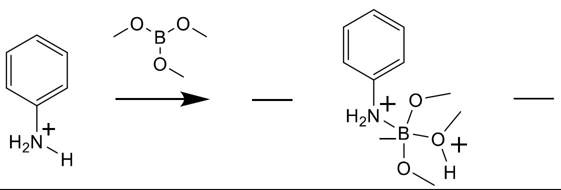	0, 1, 0
22	Proton transfer 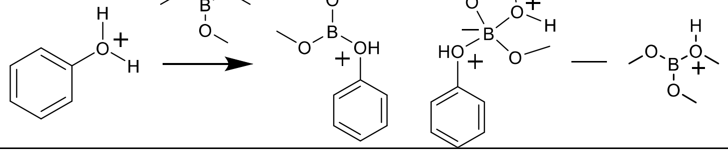	0.85, 003, 0, 1**
23	Proton transfer 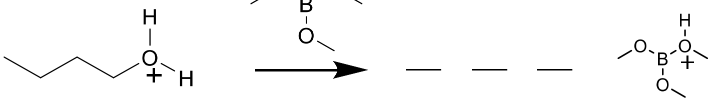	0, 0, 0, 1**
24	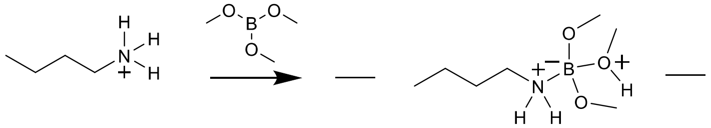	0, 1, 0
25	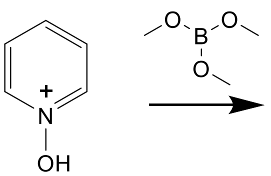 No reaction	-
26	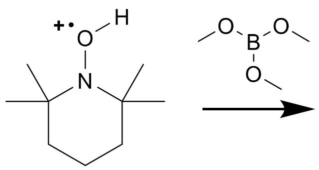 No reaction	-

* adduct - 2 methanol. ** proton transfer.

Table S7. Reactions used to train decision tree models with TDMAB neutral reagent. Diagnostic product branching ratios are shown for adduct, adduct-dimethylamine, and adduct-2 dimethylamine products, respectively. Structures of formed diagnostic product species are shown right of reaction arrows, with dashed lines indicating no diagnostic product of a given species formed.

Analyte number	Training set reactions	Diagnostic product branching ratio
1	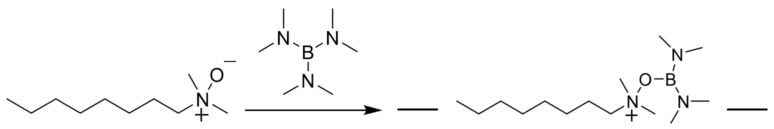	0, 1, 0
2	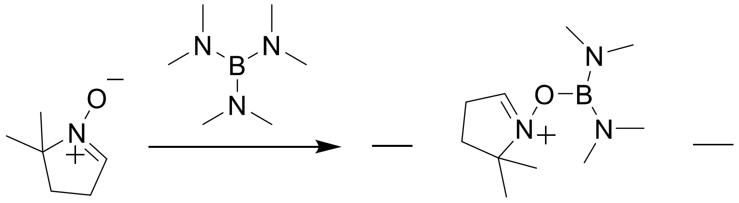	0, 1, 0
3	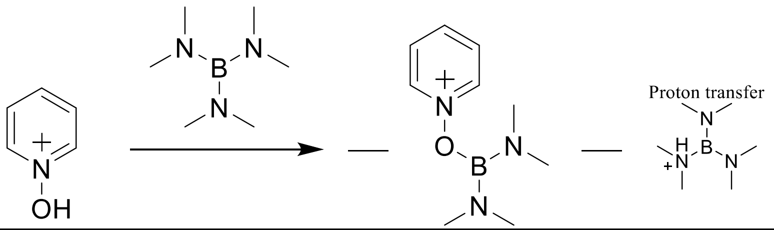	0, 0.5, 0, 0.5*
4	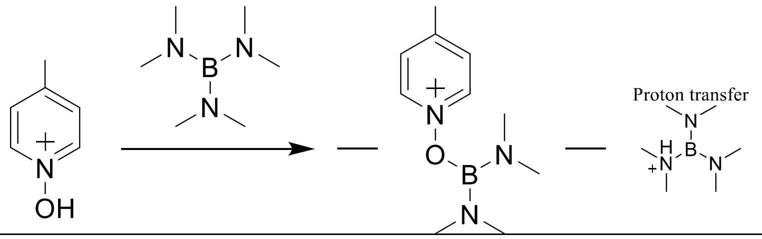	0, 0.63, 0, 0.37
5	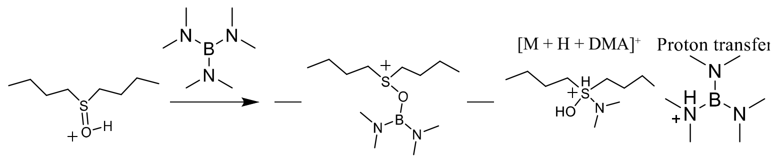	0, 0.6, 0, 0.28**, 0.12*
6	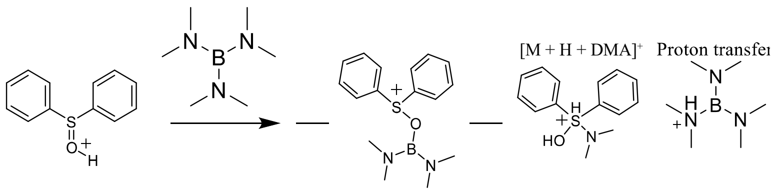	0, 0.63, 0, 0.22**, 0.15*

7	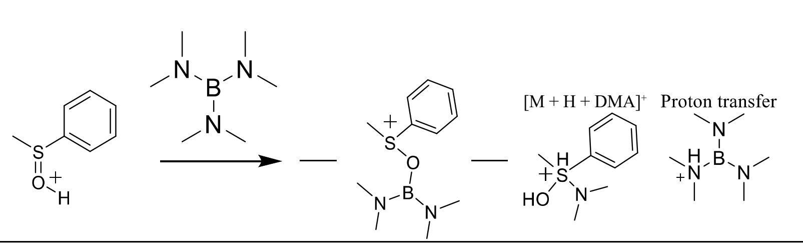	0, 0.32, 0, 0.02**, 0.66*
8	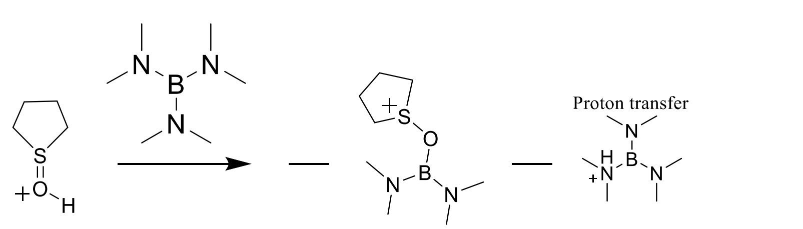	0, 0.4, 0, 0.6*
9	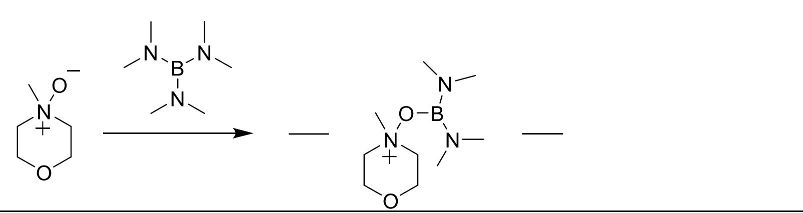	0, 1, 0
10	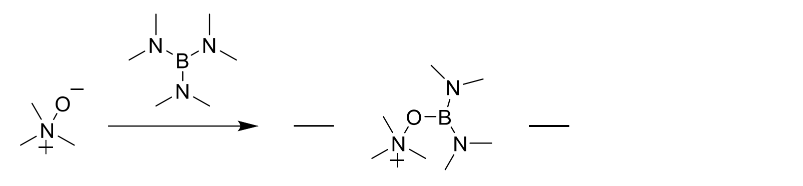	0, 1, 0
11	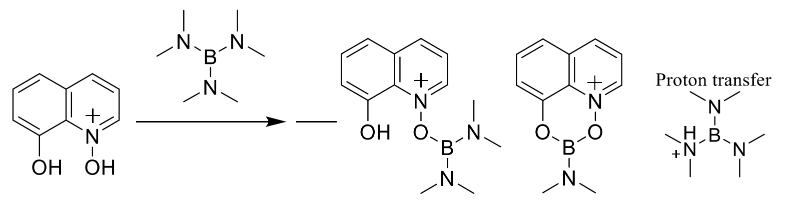	0, 0.03, 0.11, 0.86*
12	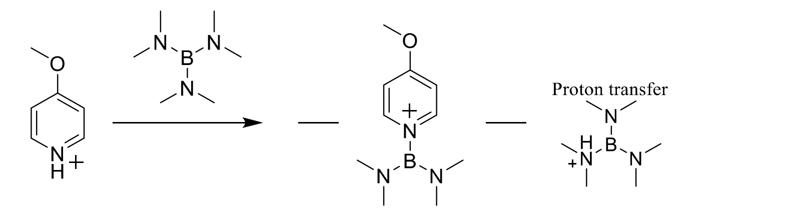	0, 0.55, 0, 0.45*
13	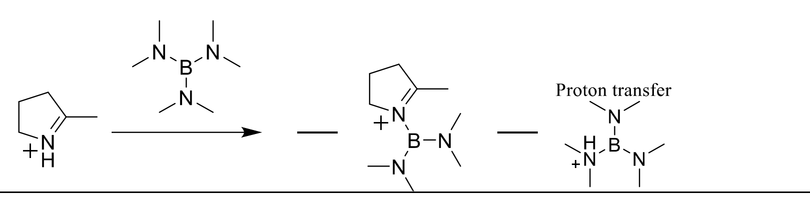	0, 0.22, 0, 0.78*

14	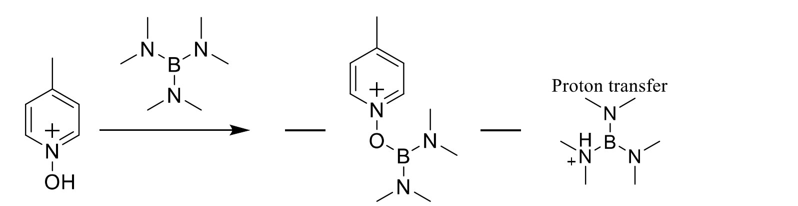	0, 0.63, 0, 0.37*
15	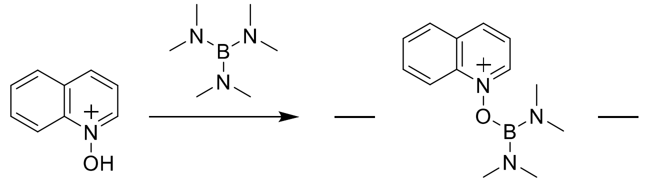	0, 1, 0
16	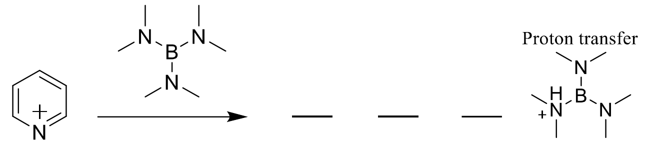	0, 0, 0, 1*
17	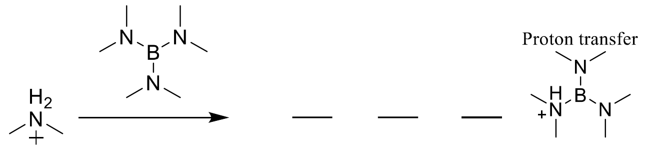	0, 0, 0, 1*
18	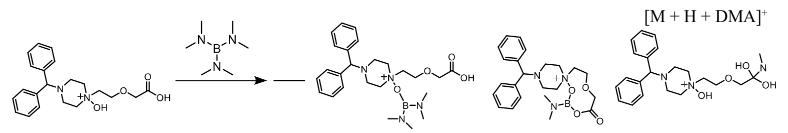	0, 0.41, 0.45, 0.14**
19	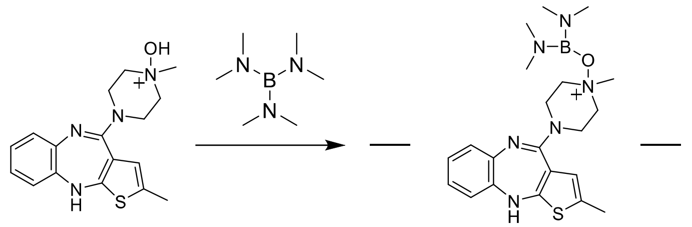	0, 1, 0
20	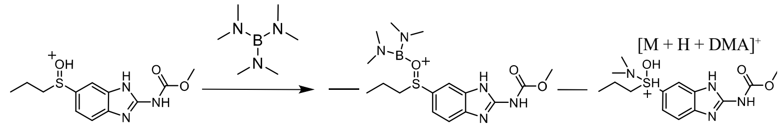	0, 0.62, 0, 0.38
21	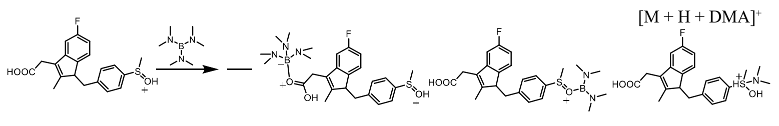	0.03, 0.93, 0, 0.04**

22	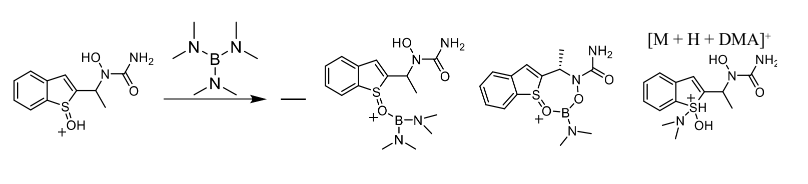	0, 0.36, 0.05, 0.59**
23	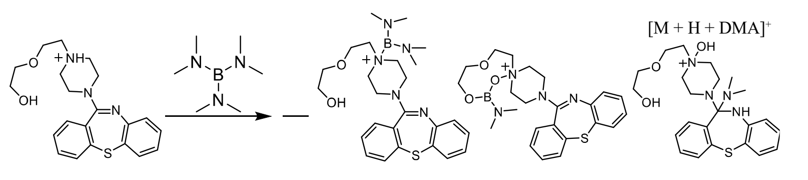	0, 0.02, 0.95, 0.03**
24	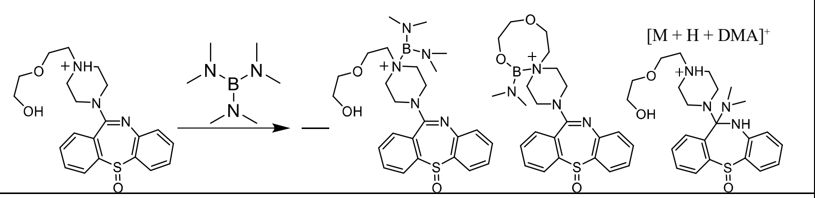	0, 0.02, 0.86, 0.12**
25	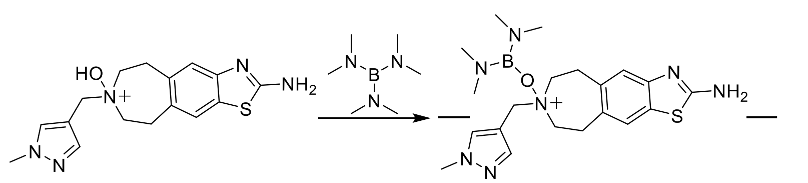	0, 1, 0
26	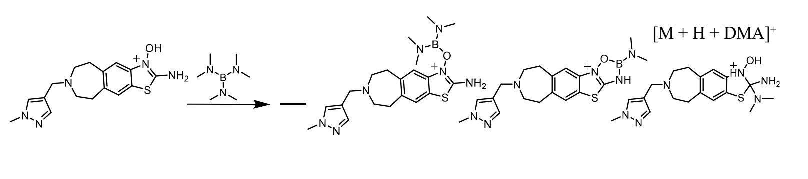	0, 0.83, 0.12, 0.05**
27	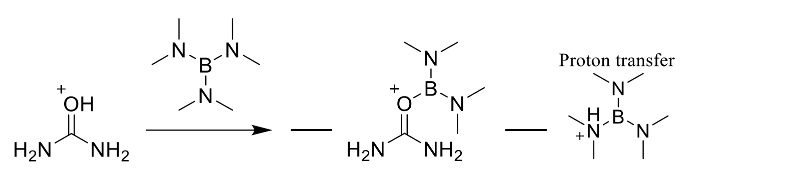	0, 0.04, 0, 0.96*
28	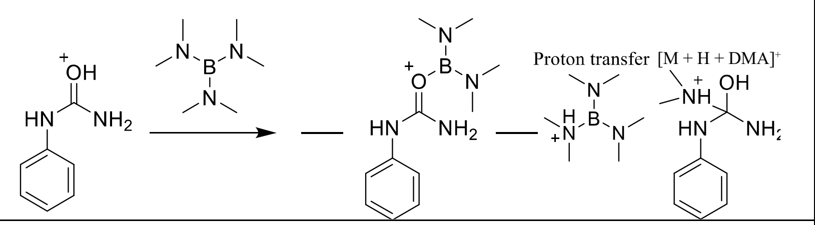	0, 0.45, 0, 0.06*, 0.49**
29	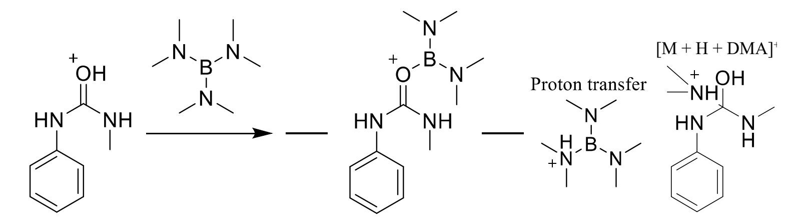	0, 0.50, 0, 0.39*, 0.11**

30	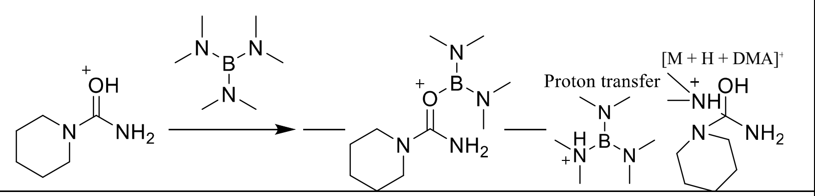	0, 0.50, 0, 0.31*, 0.14**
31	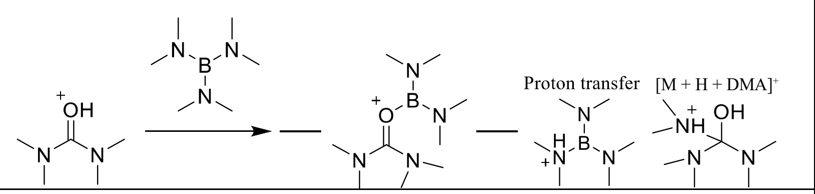	0, 0.18, 0, 0.29*, 0.53**
32	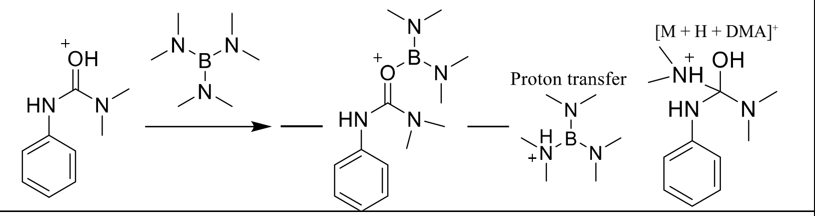	0, 0.30, 0, 0.65*, 0.05**
33	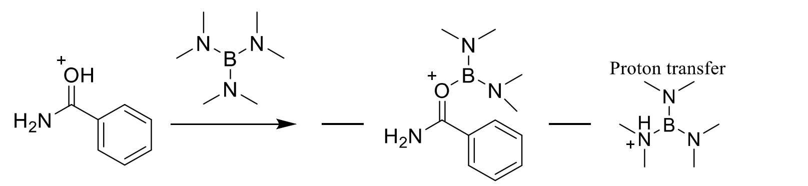	0, 0.05, 0, 0.95*
34	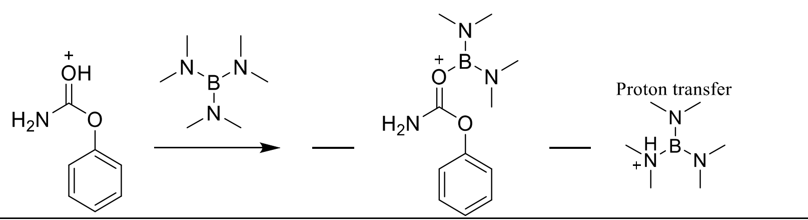	0, 0.01, 0, 0.99*
35	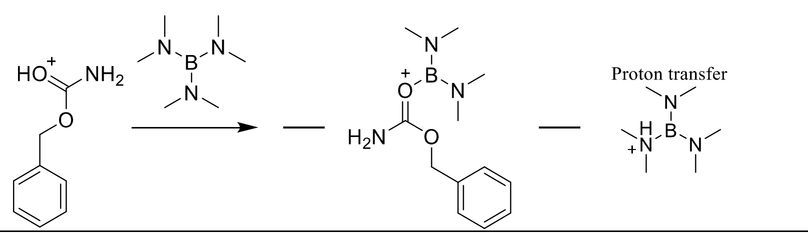	0, 0.08, 0, 0.92*

* proton transfer. ** $[M + H + DMA]^+$.

Table S8. Parameters generated by Paddy when optimizing introduction of MOP and its resolution values.

Seed	Pumping-out Time (s)	Pulsing-in Time (μs)	Fitness	Average Resolution	Resolution Peak Pair 1	Resolution Peak Pair 2	Resolution Peak Pair 3
0	1.4	120	-0.1155	2.6155	2.7048	2.5782	2.5634
1	3.5	130	-4.03	6.53	6.4346	6.8394	6.316
2	1.3	170	-0.3514	2.1486	2.4709	2.1488	1.826
3	3.2	140	-2.7844	5.2844	5.6193	5.7057	4.5282
4	3.4	160	-2.753	5.253	6.4684	4.8015	4.4891
5	3.4	150	-3.2612	5.7612	5.6559	5.4517	6.176
6	3.2	150	-2.9636	5.4636	5.3593	5.6977	5.3339
7	2.6	160	-1.7391	4.2391	4.8679	4.5335	3.3158
8	2.6	100	-1.8932	4.3932	4.3442	4.4363	4.399
9	3.1	180	-2.227	4.727	4.1507	5.5344	4.4958
10	1.6	100	-0.2972	2.2028	2.0688	2.2123	2.3272
11	2.0	100	-0.6494	3.1494	3.1167	2.7921	3.5395
12	1.4	100	-0.0825	2.4175	2.2478	2.4837	2.5209
13	1.8	180	-0.3856	2.8856	2.4744	3.1592	3.0233
14	1.3	180	-0.6295	1.8705	2.0572	1.6415	1.913
15	2.3	160	-1.6307	4.1307	3.9852	4.3818	4.0252
16	1.0	150	-0.6986	1.8014	2.0447	1.5333	1.8263
17	1.8	150	-0.4122	2.9122	2.8522	2.7619	3.1226
18	3.2	150	-2.2979	4.7979	4.7436	4.4054	5.2447
19	3.4	70	-2.7991	5.2991	4.8724	5.8886	5.1363
20	3.4	180	Peaks Disregarded	–	–	–	–
21	1.0	110	-0.9278	1.5722	1.5666	1.2855	1.8644
22	2.0	110	-0.8416	3.3416	3.5617	3.6458	2.8173
23	1.4	90	-0.8874	1.6126	1.4571	1.6257	1.7549
24	1.2	120	-0.4131	2.0869	1.9685	2.1188	2.1736
25	1.0	120	-0.8792	1.6208	1.2174	1.7356	1.9092
26	2.1	130	-0.4683	2.9683	3.5823	2.9054	2.4172

27	1.3	120	-0.428	2.072	1.8712	2.2033	2.1416
28	1.0	110	-0.6831	1.8169	2.1878	1.5336	1.7293
29	1.3	90	-0.5251	1.9749	1.8234	1.9308	2.1706
30	1.0	180	-0.8581	1.6419	1.3196	1.8633	1.7429
31	1.0	70	-0.8268	1.6732	1.3213	1.5537	2.1445
32	1.0	140	-0.8136	1.6864	1.426	1.7285	1.9047
33	1.0	110	-0.9046	1.5954	1.2796	1.9858	1.5208
34	1.4	90	-0.8797	1.6203	1.8584	1.6319	1.3707
35	1.3	80	-0.1625	2.3375	1.9512	2.5058	2.5555
36	1.3	90	-0.3377	2.1623	2.1712	2.0513	2.2643
37	2.8	140	-2.7908	5.2908	5.4363	5.4257	5.0104
38	1.6	120	-0.316	2.816	2.7782	2.8193	2.8505
39	2.2	150	-0.8701	3.3701	3.3698	3.5522	3.1884
40	2.1	160	-0.9715	3.4715	3.1498	3.5439	3.7207
41	1.1	120	-0.6252	1.8748	1.6842	2.1041	1.8361
42	1.2	70	-0.4986	2.0014	2.1774	1.926	1.9008
43	2.1	80	-1.115	3.615	3.576	3.7809	3.4881
44	1.3	110	-0.4146	2.0854	2.0269	1.8181	2.4111
45	2.6	70	-2.5223	5.0223	5.1509	5.2419	4.6741
46	1.7	100	-0.2292	2.7292	2.9706	2.9318	2.2853
47	1.6	90	-0.2884	2.7884	2.3911	3.0092	2.9648
48	1.0	110	-0.8505	1.6495	1.3899	1.5693	1.9892
49	1.0	80	-0.8803	1.6197	1.2311	1.9107	1.7173
50	1.0	70	-0.7597	1.7403	1.8007	1.9406	1.4796
51	1.0	130	-0.8173	1.6827	1.8416	1.8168	1.3897
52	1.8	90	0.486	2.986	2.9753	2.8599	3.1227
53	1.5	80	0.0416	2.5416	2.8581	2.1962	2.5704
54	1.2	70	-0.7219	1.7781	1.8915	1.4354	2.0073
55	2.0	90	0.5209	3.0209	3.5219	2.7057	2.835
56	1.3	80	-0.4647	2.0353	2.1576	2.1972	1.7512

57	1.4	180	-0.8699	1.6301	1.392	1.6502	1.848
58	2.3	110	1.2392	3.7392	3.4984	3.73	3.989
59	1.3	160	-0.4755	2.0245	2.2471	2.0604	1.7661
60	1.9	100	0.7799	3.2799	3.2729	3.3472	3.2195
61	1.4	70	-0.03	2.47	2.7117	2.5654	2.133
62	1.6	80	0.199	2.699	2.7476	2.6852	2.6643

Table S9. Parameters generated by Paddy when optimizing introduction of TMB and its resolution values.

Seed	Pumping-out Time (s)	Pulsing-in Time (μs)	Fitness	Average Resolution	Resolution Peak Pair 1	Resolution Peak Pair 2	Resolution Peak Pair 3
0	2.5	140	-1.4679	3.9679	4.0318	3.9298	3.9422
1	3.5	130	-3.068	5.568	5.4238	5.5433	5.737
2	3.0	180	-2.3315	4.8315	4.3969	4.8628	5.2349
3	3.1	130	-2.3198	4.8198	4.2787	5.4947	4.686
4	2.0	120	-0.4944	2.9944	3.0144	2.7912	3.1775
5	3.8	90	-3.8838	6.3838	6.2427	6.2408	6.6679
6	1.2	120	-0.6647	1.8353	1.6859	1.8534	1.9665
7	3.2	180	-2.4913	4.9913	4.6105	5.0365	5.3268
8	3.2	140	-1.984	4.484	4.994	4.536	3.9219
9	3.4	160	-3.0421	5.5421	5.4397	5.9687	5.2179
10	1.6	90	-0.2669	2.2331	2.1916	2.1948	2.3131
11	1.3	150	-0.4097	2.0903	1.808	2.3417	2.121
12	2.5	110	-1.5023	4.0023	3.8972	4.063	4.0465
13	2.3	110	-0.5461	3.0461	3.098	3.2613	2.7791
14	1.0	90	Peaks Disregarded	–	–	–	–
15	1.5	110	-0.2026	2.2974	2.4433	2.337	2.1119
16	2.3	120	-0.7452	3.2452	3.4046	3.6113	2.7197
17	2.2	120	-0.8952	3.3952	4.2297	3.0741	2.8817
18	3.7	150	-3.0022	5.5022	5.8631	5.4961	5.1475
19	1.4	110	-0.543	1.957	1.8114	1.6603	2.3992
20	1.9	120	-0.5167	3.0167	2.9133	2.6303	3.5066
21	1.0	110	-0.9011	1.5989	1.8922	1.3896	1.5148
22	1.1	70	Peaks Disregarded	–	–	–	–
23	1.6	70	Peaks Disregarded	–	–	–	–
24	2.4	90	Peaks Disregarded	–	–	–	–

25	1.4	70	Peaks Disregarded	–	–	–	–
26	2.3	90	Peaks Disregarded	–	–	–	–
27	1.2	170	-0.8143	1.6857	1.7878	1.7139	1.5552
28	2.3	140	-0.5015	3.0015	2.4062	2.773	3.8255
29	1.9	100	-0.5582	3.0582	3.7704	2.7906	2.6138
30	2.4	100	-0.8555	3.3555	2.4003	3.5246	4.1417
31	1.9	110	-0.3734	2.8734	2.5546	2.7252	3.3405
32	2.4	130	-1.3772	3.8772	3.9533	3.8855	3.7928
33	1.5	140	-0.4452	2.0548	1.8904	2.1174	2.1565
34	1.4	140	-0.1596	2.3404	2.4547	2.3717	2.1947
35	1.6	110	-0.9422	1.5578	1.6768	1.505	1.4917
36	1.5	110	-0.3066	2.8066	2.4816	2.9688	2.9694
37	1.3	70	Peaks Disregarded	–	–	–	–
38	1.7	180	Peaks Disregarded	–	–	–	–
39	2.5	130	-1.1378	3.6378	3.846	3.397	3.6703
40	2.0	150	-1.142	3.642	3.5299	3.6876	3.7085
41	1.3	110	-0.9336	1.5664	1.5825	1.3931	1.7237
42	1.0	180	-0.9553	1.5447	1.3236	1.8454	1.4651
43	1.0	130	-0.864	1.636	1.6482	1.8827	1.3771
44	1.0	100	-0.9928	1.5072	1.1376	1.4299	1.9539
45	1.5	140	-0.2886	2.2114	2.4417	2.3236	1.8689
46	1.7	90	-0.247	2.747	3.0636	2.3359	2.8416
47	2.1	110	-0.9553	3.4553	2.954	4.1717	3.2403
48	2.1	80	Peaks Disregarded	–	–	–	–
49	2.5	100	Peaks Disregarded	–	–	–	–
50	1.3	90	Peaks Disregarded	–	–	–	–

51	2.3	130	-0.9959	3.4959	4.1593	3.354	2.9744
52	2.6	130	1.2896	3.7896	3.9346	3.7131	3.721
53	2.2	110	0.9111	3.4111	3.7374	3.4319	3.0639
54	1.0	150	-0.7425	1.7575	1.9922	1.4995	1.7807
55	1.6	120	0.2681	2.7681	2.997	2.9306	2.3768
56	1.5	110	-0.3862	2.1138	1.9838	2.2777	2.08
57	1.7	150	0.2744	2.7744	2.9377	2.6166	2.7687
58	1.9	110	0.03	2.53	3.0669	2.1831	2.34
59	1.7	100	0.0985	2.5985	2.6708	2.6629	2.4618
60	2.0	70	Peaks Disregarded	–	–	–	–

Table S10. Parameters generated by Paddy when optimizing introduction of TDMAB and its resolution values.

Seed	Pumping-out Time (s)	Pulsing-in Time (μs)	Fitness	Average Resolution	Resolution Peak Pair 1	Resolution Peak Pair 2	Resolution Peak Pair 3
0	2.4	110	-0.3629	2.1371	2.2186	2.1222	2.0705
1	2.2	120	-0.6935	1.8065	1.7691	1.9462	1.7043
2	2.8	150	-0.4139	2.0861	2.0557	2.0534	2.1492
3	2.3	180	-0.5968	1.9032	1.9671	2.0579	1.6847
4	1.8	180	-0.9132	1.5868	1.6256	1.746	1.3889
5	1.8	180	-1.3086	1.1914	1.1075	1.1495	1.3171
6	1.4	180	-1.5102	0.9898	1.2039	0.929	0.8364
7	3.3	180	-0.2003	2.2997	2.4646	2.0549	2.3795
8	1.2	120	-1.2962	1.2038	1.1802	1.0573	1.3739
9	1	130	-1.5896	0.9104	0.9707	0.8138	0.9468
10	3.4	160	-0.064	2.436	2.2757	2.2032	2.8291
11	3.4	170	-0.1218	2.6218	2.3602	2.6991	2.8063
12	4	180	-0.2211	2.7211	2.7902	2.7383	2.6349
13	2.5	110	-0.3894	2.1106	2.2084	2.1208	2.0027
14	1.7	120	-1.0002	1.4998	1.529	1.4995	1.4708
15	3.6	150	-0.2905	2.7905	3.0385	2.6115	2.7214
16	2.7	100	-0.1259	2.3741	2.589	2.3598	2.1736
17	3	160	-0.1117	2.3883	2.2301	2.4447	2.4902
18	3	180	-0.209	2.291	2.3902	2.4116	2.0712
19	1.4	180	-1.3301	1.1699	1.1285	1.1703	1.2109
20	3.5	170	-0.8333	3.3333	3.5693	3.7379	2.6928
21	1.6	120	-1.0783	1.4217	1.3417	1.3931	1.5303
22	3.4	120	0.7924	3.2924	3.2367	3.3095	3.331
23	3	170	-0.0446	2.4554	2.5492	2.3974	2.4195
24	3.4	110	0.598	3.098	3.0798	3.1151	3.0991
25	4	170	0.7829	3.2829	3.2383	3.2932	3.3173
26	2.7	180	-0.4008	2.0992	2.3312	2.0154	1.951

27	3.7	130	0.8975	3.3975	3.447	3.3056	3.44
28	3.7	160	0.5314	3.0314	3.046	2.9275	3.1207
29	3.4	140	0.5114	3.0114	3.1333	2.9817	2.9193
30	3.5	160	0.4319	2.9319	3.0376	2.7941	2.964
31	3.1	110	0.5584	3.0584	2.9193	3.2112	3.0448

Table S11. Experimentally identified neutral reagents that can undergo diagnostic ion-molecule reactions with the protonated analytes shown below. Machine learning based predictions are shown in the last column.

Analyte	Experimentally identified neutral reagents			x
	MOP	TMB	TDMAB	
Diphenyl sulfoxide	✓	✓	✗	MOP, TMB
Methyl phenyl sulfone	✗	✓	✗	TMB
Pyridine N-oxide	✓	✗	✓	MOP, TDMAB

Table S12. Test set diversity of each neutral reagent model

Neutral Reagent	Diagnostic Product	Test Set – Average Pairwise Tanimoto Similarity
TDMAB	Adduct-DMA	0.0649
TDMAB	Adduct-2DMA	0.0649
TMB	Adduct	0.3009
TMB	Adduct-Me2O	0.3634
TMB	Adduct-MeOH	0.3344

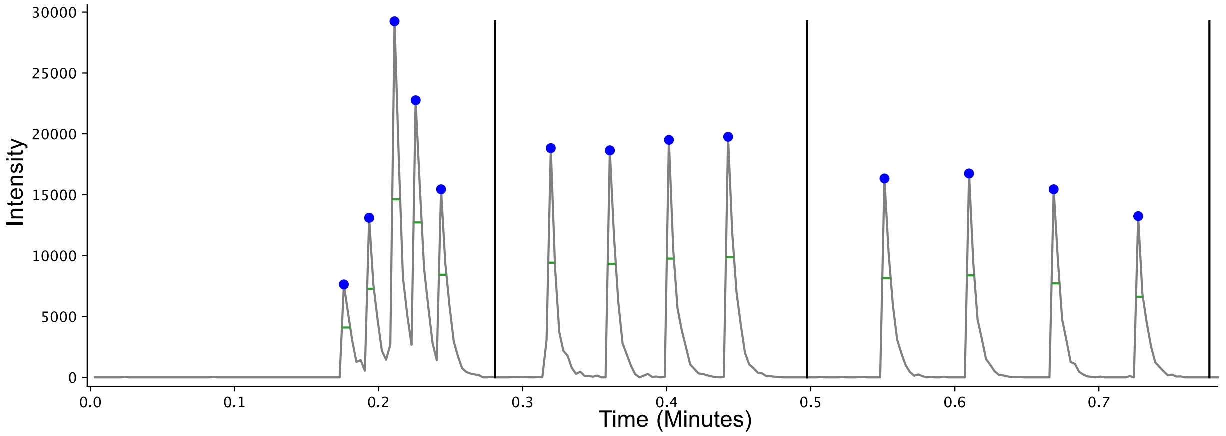

Figure S1. Truncated extracted ion profile as a function of experiment time for the zeroth (initiation) iteration of the optimization of pulsing-in and pumping-out times for TDMAB. Displayed are the initial five tuning peaks and two subsequent Paddy generated peak clusters. The blue dots depict the identified times for peak maxima and green lines as the width of the peaks at the half height. Black vertical lines display the anticipated midpoints between peak clusters calculated using the pumping-out times from the recipe file. Midpoints are used to segment extracted ion profiles such to allow association of programed pulsing-in of reagents to their resulting fitness/resolution.

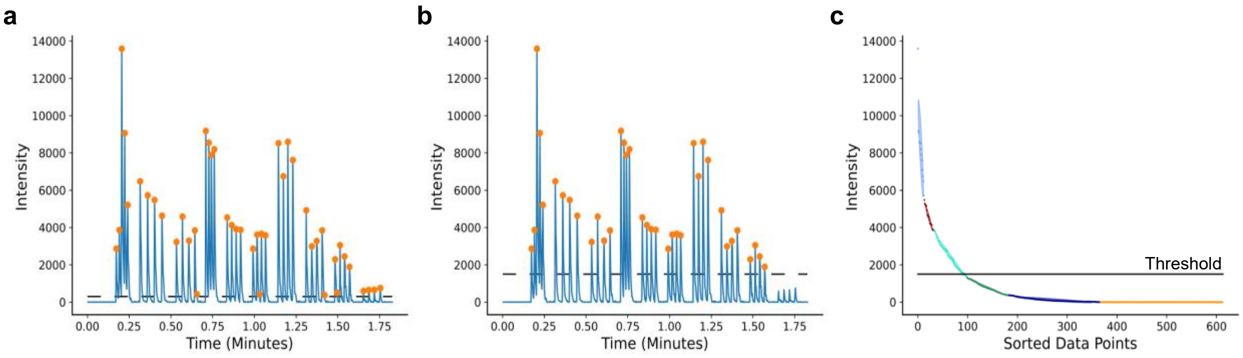

Figure S2. Overview of data dependent peak selection via Gaussian mixture models (GMMs), with the extracted ion profile being the fifth iteration of the optimization of pulsing-in and pumping-out times for TMB. **a**) Extracted ion profile as a function of experiment time with peaks identified by the SciPy 'find_peaks' function indicated by orange dots while using an arbitrary peak height threshold of 300. **b**) Extracted ion profile as a function of experiment time with peaks identified based on the GMM based threshold. Noise peaks and peaks programed by Paddy-PUMP with insufficient height have not been selected for time domain orientation or resolution calculations. **c**) Datapoints selected from the TBM extracted ion profile and sorted from high to low **value**, and clustered using a GMM. Clusters are colored separately, with the GMM intensity threshold defined in the methods section in main text (**eq. 3**).

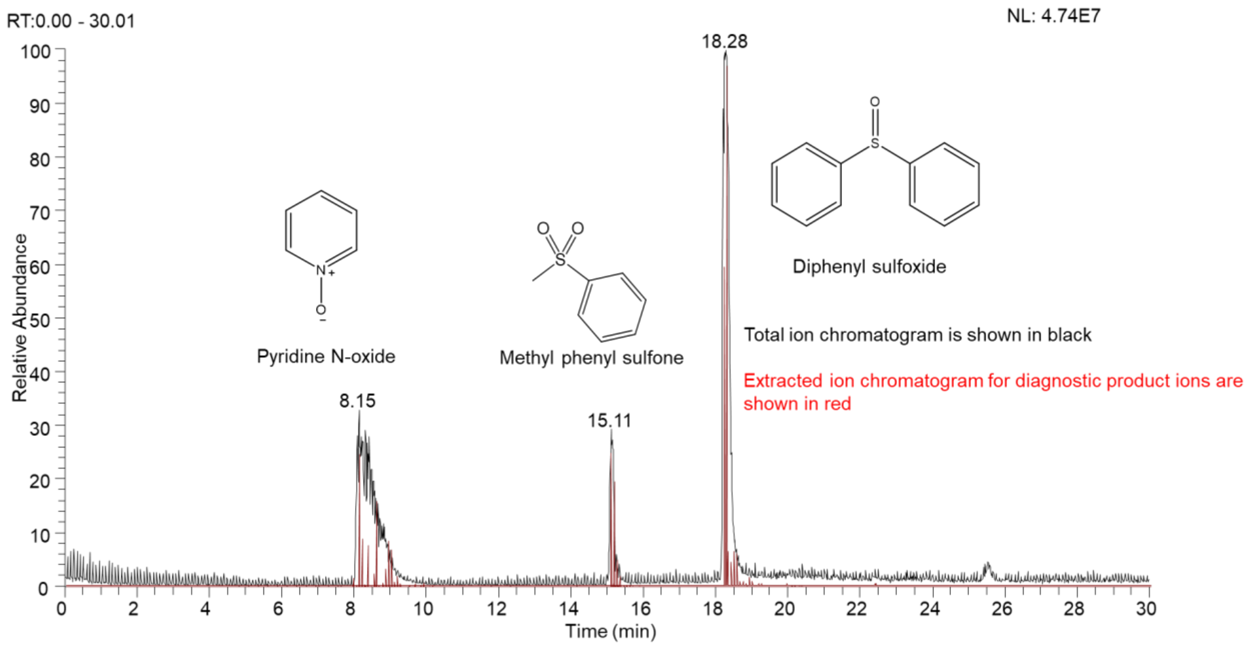

Figure S3. HPLC total ion chromatogram and extracted ion chromatogram for diagnostic product ions measured for a mixture of three model compounds at a concentration of 2 mg/mL in methanol

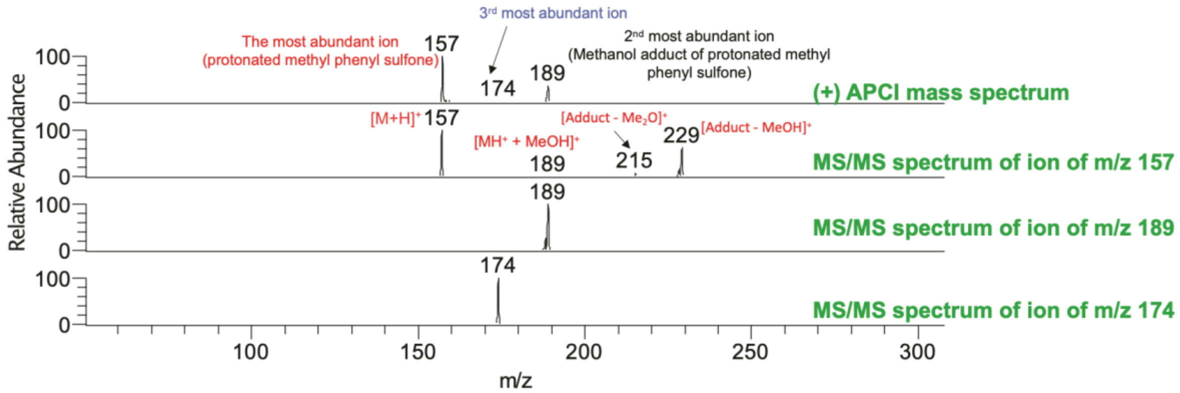

Figure S4. MS/MS spectra measured after 30 ms ion-molecule reactions with TMB for the three most abundant ions generated upon APCI from methyl phenyl sulfone as it eluted from the HPLC. Spectra showing no selective ion-molecule reactions of protonated methyl phenyl sulfone with TDMAB and MOP are provided in **Figure S9**.

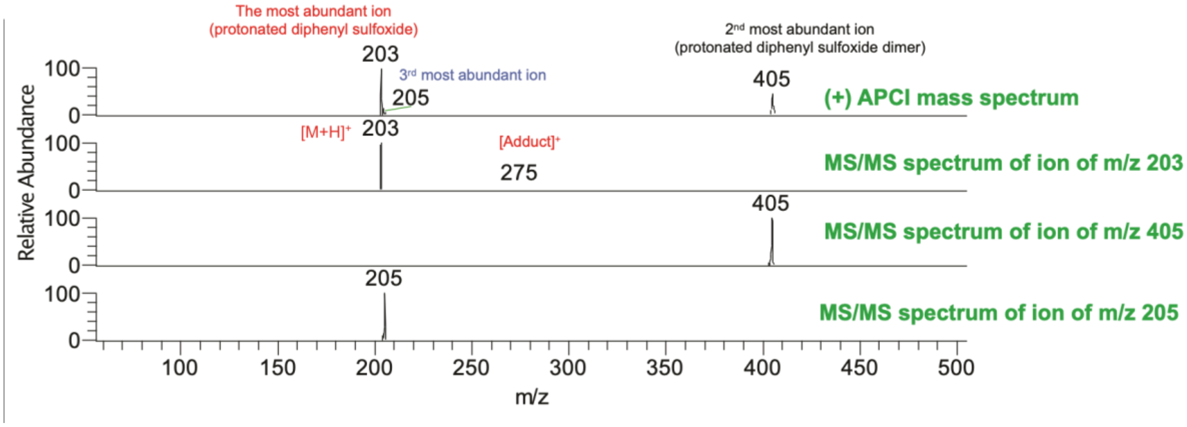

Figure S5. MS/MS spectra measured after 30 ms ion-molecule reactions with MOP for the three most abundant ions generated upon APCI from diphenyl sulfoxide as it eluted from the HPLC.

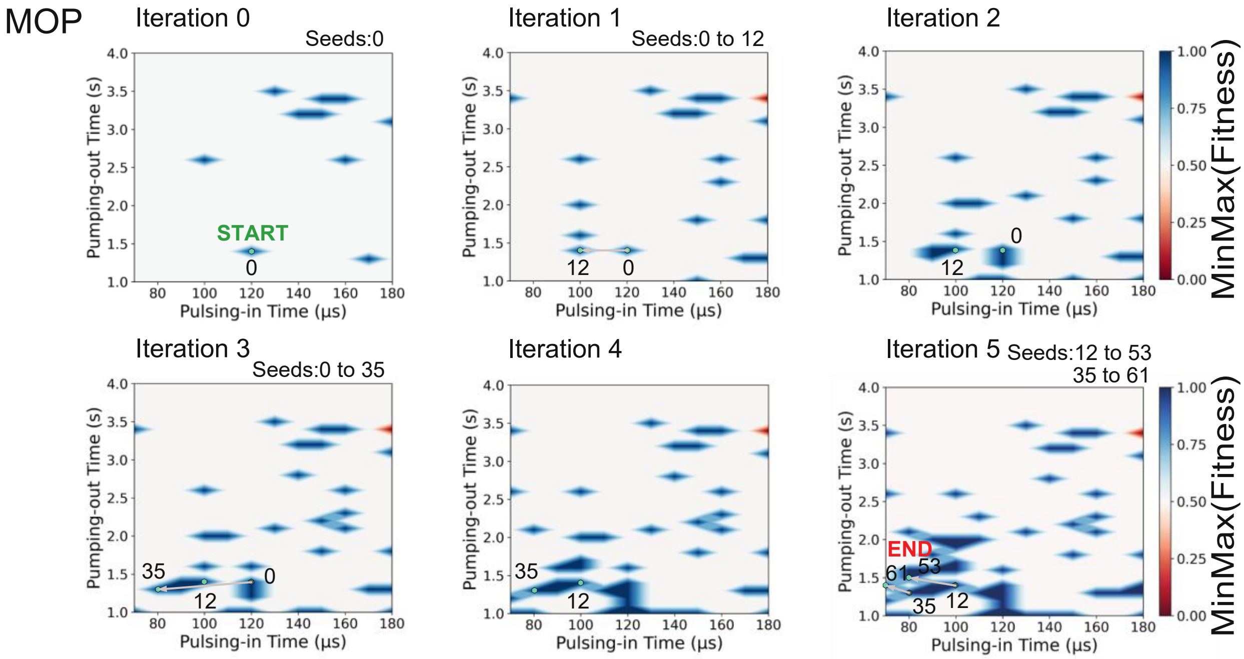

Figure S6. Contour maps displaying MOP optimization with Paddy-PUMP over six iterations. Evaluated parameters (Paddy plants) involved in yielding the solutions for the experiment are displayed as green dots with their seed number (Table S8) displayed beside them. Plants from prior iterations are displayed as a darker hue of green, and an arrow points from them to their progeny (new parameters generated via sampling a distribution centered on the prior parameters). If a plant does not produce a seed directly involved in generating one of the optimized sets of pulsing-in and pumping-out times during an iteration it remains bright green with no arrow until the iteration in which it does.

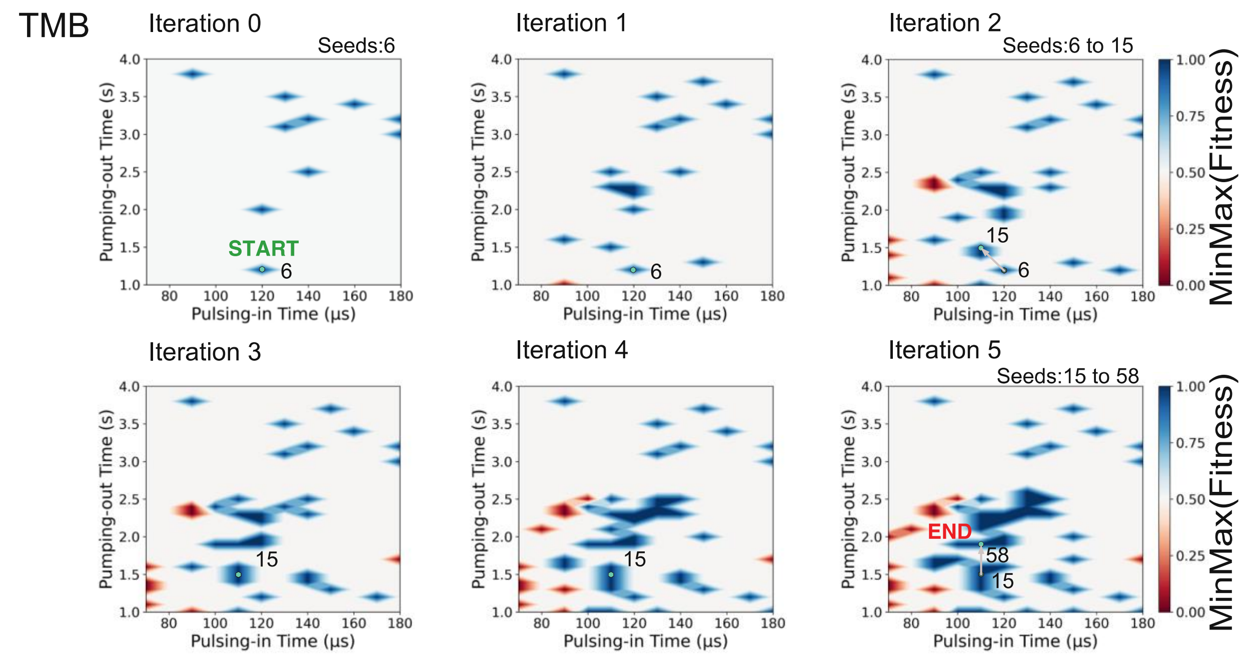

Figure S7. Contour maps displaying TMB optimization with Paddy-Pump over six iterations. Fitness values are min-max normalized to range from zero to one and displayed using a divergent blue-red color scale. Evaluated parameters (Paddy plants) involved in yielding the solution for the experiment are displayed as green dots with their seed number (Table S9) displayed beside them. Plants from prior iterations are displayed as a darker hue of green, and an arrow points from them to their progeny (new parameters generated via sampling a distribution centered on the prior parameters). If a plant does not produce a seed directly involved in generating the optimized set of pulsing-in and pumping-out times during an iteration it remains bright green with no arrow until the iteration it does.

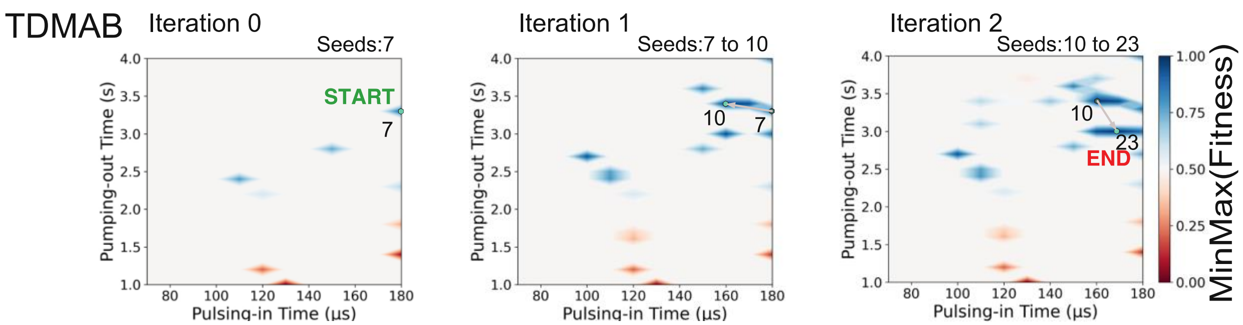

Figure S8. Contour maps displaying TDMAB optimization with Paddy-Pump over three iterations. Fitness values are min-max normalized to range from zero to one, and displayed using a divergent color blue-red scale. Evaluated parameters (Paddy plants) involved in yielding the solution for the experiment are displayed as green dots with their seed number (Table S10) displayed beside them. Plants from prior iterations are displayed as a darker hue of green, and an arrow points from them to their progeny (new parameters generated via sampling a distribution centered on the prior parameters).

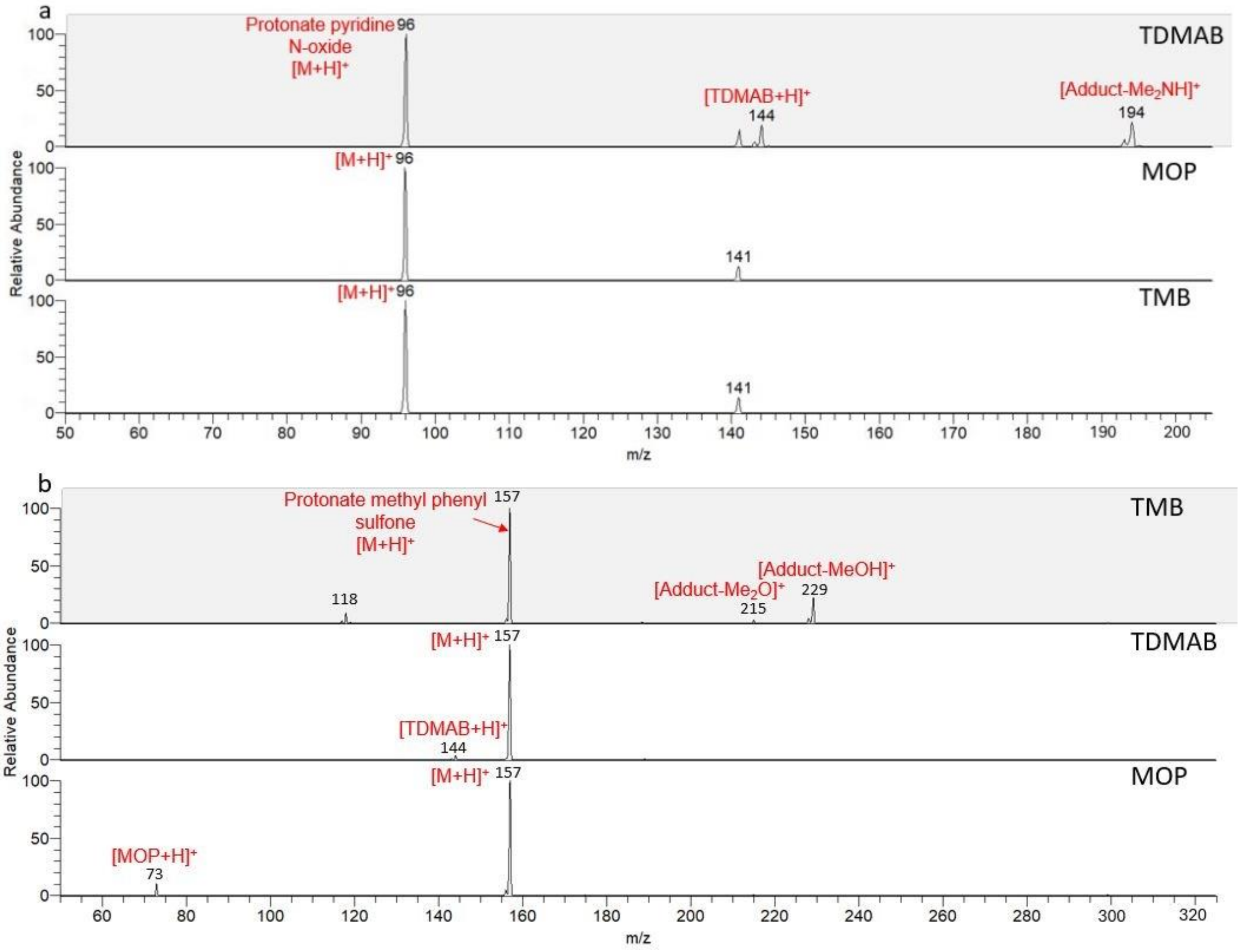

Figure S9. a) Mass spectra showing a selective ion-molecule reaction between protonated pyridine N-oxide and TDMAB, alongside spectra demonstrating no selective reaction with MOP or TMB. b) Mass spectra illustrating a selective ion-molecule reaction between protonated methyl phenyl sulfone and TMB, with no reaction observed for protonated methyl phenyl sulfone with TDMAB or MOP.

Section S1. Three Case Studies Linking Decision Paths to Chemical Rationale

To further demonstrate interpretability of the machine-learning models, we provide three representative case studies, one each for MOP, TMB, and TDMAB-showing how an individual decision path in a diagnostic-product tree maps to a recognizable structural motif and ultimately to a functional-group assignment. These examples correspond directly to the analytes illustrated in Figure 5a.

This section illustrates how the full functional-group identification module operates on real diagnostic ion–molecule reaction mass spectra for the three analytes shown in Figure 5a–b. These examples reflect the actual implementation in the provided Python script.

For each analyte–reagent pair, the module takes as inputs:

- The full mass spectrum measured after the diagnostic ion–molecule reaction
- The protonated analyte m/z (analyte_mz)
- A user-defined relative-intensity cutoff (relative_cutoff), used for selecting peaks for branching-ratio calculations
- The analyte's elemental composition (elem_comp)
- The analyte's RDBE (rdbe)

How the module computes mass differences (as implemented)

After reading the reaction spectrum, the script:

- Sorts peaks by intensity and normalizes intensities.
- Applies the user-specified relative-intensity cutoff.
- Removes the protonated analyte peak.
- For every remaining peak above the cutoff, computes the mass difference:

$$\Delta m = \left(m/z_{product\ ion}\right) - \left(m/z_{analyte}\right)$$

These computed mass differences (not preset) values are then mapped to reagent-specific diagnostic channels for both expert-based and ML-derived functional groups.

The functional-group identification module operates directly on the experimental ion-molecule reaction mass spectrum. After reading the spectrum, the module normalizes peak intensities, applies a user-defined relative-intensity cutoff, and removes the precursor ion corresponding to the protonated analyte. For every remaining peak above the cutoff, the module calculates a mass difference by subtracting the analyte's m/z from the product-ion m/z. These mass differences are then matched against reagent-specific expert-based diagnostic channels and ML-derived diagnostic-product fragments obtained from the decision-tree models. Both the expert-based and ML-based candidate functional groups are subsequently filtered using the analyte's measured elemental composition and RDBE, retaining only those functionalities that are chemically feasible for that specific elemental formula. The module then reports the remaining functional groups as two parallel predictions, one derived from expert reaction heuristics and the other derived from ML-identified structural motifs as illustrated in Figure 5a.

<u>Case Study 1 - MOP: Identification of a Sulfoxide in Diphenyl Sulfoxide</u>

Inputs

Analyte's measured m/z 203
Relative cutoff = 0.01
Measured elemental composition = $C_{12}H_{11}O_1S_1$
Measured RDBE = 7.5

Neutral reagent: MOP
Observed diagnostic product ions: MOP adduct ((m/z 275) +72 from the m/z of the isolated protonated compound (m/z 203))
Reference: Figure 5a (top row), Figure 6d

Decision-tree path (simplified):

1. Fingerprint bit corresponding to an S=O (sulfoxide) environment is present.
2. Bit associated with an aromatic substituent adjacent to sulfur is present.
3. Bits characteristic of tertiary N-oxides are absent.

DT Interpretation:
The decision-tree branch identifies an oxygenated sulfur center embedded in an aromatic system - consistent with a sulfoxide and inconsistent with tertiary N-oxides or other heteroatom motifs.

Chemical rationale:
MOP is well-known to selectively form stable adducts with protonated sulfoxides due to oxygen nucleophilicity and restricted overoxidation pathways. The analyte shows:

- presence of ions of +72 m/z units from the protonated analyte
- an elemental composition containing S and O,
- an aromatic RDBE pattern consistent with diphenyl sulfoxide, and
- decision-tree bits that map to known MOP sulfoxide reactivity

The obtained elemental composition, RDBE value, the presence of ions of +72 m/z units from the protonated analyte, and the decision-tree path reinforce the assignment of an aromatic sulfoxide.

Module workflow:

The code reads the MOP reaction spectrum, applies a relative-intensity cutoff of 0.01, removes the analyte peak (m/z 203), and computes mass differences for all remaining peaks above the cutoff. One of these peaks produces a mass difference that falls within ±1.04 Da of the known MOP diagnostic channel at 72.06 Da. Then the module picks the best matching functional groups that aligns with the input elemental composition, RDBE, and the calculated mass difference of 72.06 Da. Then module gives out the best predictions using ML-based and expert-based functional groups.

Predicted functional-group output (**Figure 5a**): Sulfoxide (ML-based and expert-based predictions are in concordance)

<u>Case Study 2 - TMB: Identification of a Sulfone in Methyl Phenyl Sulfone</u>

Inputs

Analyte's measured m/z: 157

Relative cutoff: 0.001 (TMB conditions)
Measured elemental composition: $C_7H_9O_2S_1$
Measured RDBE: 3.5
Neutral reagent: TMB
Observed diagnostic product ions: TMB adduct - Me_2O ((m/z 215 at +58 from the m/z of the isolated protonated compound (m/z 157))
Reference: Figure 5a (middle row), Figure 6b

Decision-tree path (simplified):

1. Fingerprint bit corresponding to a tetrahedral sulfur(VI) (SO_2) environment is present.
2. Bits associated with adjacent heteroatoms that might indicate sulfoxides or other competing functionalities are absent.
3. A bit identifying aryl substitution adjacent to the sulfur center is present.

DT Interpretation:
The combination of a strongly oxygenated sulfur center (SO_2) with an sp^2-substituted carbon environment is characteristic of sulfones. The absence of competing functionalities (e.g., sulfoxides) steers the prediction toward sulfone.

Chemical rationale:
TMB readily undergoes proton transfer reactions with protonated sulfones to form an adduct followed by loss of a Me_2O molecule, generating characteristic neutral-loss or adduct signals. The m/z 215 at +58 m/z units from the protonated analyte is a diagnostic product and the decision-tree branch illustrates that the model pinpoints the sulfone oxygen-transfer reactivity motif.

- A prominent ion at m/z 215 corresponding to +58 Da.
- An elemental composition containing S and O_2.
- An RDBE consistent with an aryl sulfone.
- Decision-tree bits that match known TMB reactivity toward sulfones.

Module workflow:

The module processes the TMB reaction spectrum by normalizing peak intensities, applying the TMB-specific relative-intensity cutoff (0.001), and removing the precursor ion at m/z 157. It then computes mass differences for every remaining peak above the cutoff. A strong peak produces a mass difference falling within ±1.04 Da of the known TMB diagnostic channel at 59.03 Da (TMB adduct-Me_2O). Using this mass difference, the module queries both the expert-based and ML-based TMB dictionaries and filters all candidate functionalities using the analyte's elemental composition and RDBE. Only functional groups compatible with the SO_2 motif, the mass difference, and the analyte's formula remain.

Predicted functional-group output (**Figure 5a**): Sulfone (both ML-based and expert-based predictions agree)

Case Study 3 - TDMAB: Identification of an N-Oxide in Pyridine N-Oxide

Inputs

Analyte's measured m/z: 96
Relative cutoff: 0.01
Measured elemental composition: $C_5H_6N_1O_1$
Measured RDBE: 3.5
Neutral reagent: TDMAB
Observed diagnostic product ions: ((m/z 194) +98 from the m/z of the isolated protonated compound (m/z 96)) TDMAB adduct - DMA
Reference: Figure 5a (bottom row), Figure 6c

Decision-tree path (simplified):

1. Fingerprint bit corresponding to an N-O oxidation motif characteristic of N-oxides is present.
2. Fingerprint bit indicating aromatic heterocycle present
3. No signal for sulfur-oxygen motifs (e.g., sulfoxides or sulfones)

DT Interpretation:

The tree distinguishes N-oxide and sulfoxide motifs by the absence of sulfur-related fingerprint bits and the presence of an aromatic nitrogen environment. The presence of the N-O functionality is recognized as the key determinant for reactivity with TDMAB.

Chemical rationale:

TDMAB selectively forms highly stable adducts that lose DMA as a neutral molecule with heteroaromatic N-oxides. The decision-tree path mirrors this reagent-specific reactivity pattern and yields an N-oxide prediction consistent with the observed mass difference.

- A strong diagnostic ion at m/z 194 (+98 Da).
- An elemental composition requiring N and O.
- An aromatic RDBE consistent with a pyridine N-oxide structure.
- Decision-tree bits uniquely aligned with TDMAB reactivity toward N-oxides.

Predicted functional-group output (**Figure 5a**): N-Oxide (ML-based and expert-based predictions agree)
